# Multi-strategic RNA-seq analysis reveals a high-resolution transcriptional landscape in cotton

**DOI:** 10.1038/s41467-019-12575-x

**Published:** 2019-10-17

**Authors:** Kun Wang, Dehe Wang, Xiaomin Zheng, Ai Qin, Jie Zhou, Boyu Guo, Yanjun Chen, Xingpeng Wen, Wen Ye, Yu Zhou, Yuxian Zhu

**Affiliations:** 1 College of Life Sciences, Wuhan University, 430072 Wuhan, Hubei China; 2 State Key Laboratory of Virology, Wuhan University, 430072 Wuhan, Hubei China; 3 Institute for Advanced Studies, Wuhan University, 430072 Wuhan, Hubei China; 4 Medical Research Institute, School of Medicine, Wuhan University, 430072 Wuhan, Hubei China

**Keywords:** Genome informatics, Gene expression, Transcriptomics, Plant development

## Abstract

Cotton is an important natural fiber crop, however, its comprehensive and high-resolution gene map is lacking. Here we integrate four complementary high-throughput techniques, including Pacbio long read Iso-seq, strand-specific RNA-seq, CAGE-seq, and PolyA-seq, to systematically explore the transcription landscape across 16 tissues or different organ types in *Gossypium arboreum*. We devise a computational pipeline, named IGIA, to reconstruct accurate gene structures from the integrated data. Our results reveal a dynamic and diverse transcriptional map in cotton: tissue-specific gene expression, alternative usage of TSSs and polyadenylation sites, hotspot of alternative splicing, and transcriptional read-through. These regulated events affect many genes in various aspects such as gain or loss of functional RNA motifs and protein domains, fine-tuning of DNA binding activity, and co-regulation for genes in the same complex or pathway. The methods and findings provide valuable resources for further functional genomic studies such as understanding natural SNP variations for plant community.

## Abstract (web summary)

In-depth functional characterization of genomes relies on comprehensive transcriptome data. Here, the authors employ four complementary RNA sequencing technologies to explore the transcription landscape across 16 tissues or different organ types in diploid A genome cotton using a newly developed computational pipeline.

## Introduction

Cotton fiber, an excellent model for studying cell elongation and cell wall biosynthesis, is the principal natural source for textile industry. Thus, cotton is one of the most important fiber crop plants world-wide and has long been one of major focuses of plant research^1^. Recently, four different cotton genomes have been successfully sequenced and assembled, including two allotetraploid (AADD) *Gossypium hirsutum*^2–4^, and *Gossypium barbadense*^3,4^ genomes, and two ancestor diploid *Gossypium raimondii* (DD)^1,5^ and *Gossypium arboreum* (AA)^6,7^ genomes. In recent years, transcriptomic profiling analysis has also been reported for a close relative of *G. arboreum*, Levant cotton (*Gossypium herbaceum*)^8^.

The heterogeneity of RNAs produced from different promoter usage, alternative splicing (AS) and polyadenylation significantly increases the transcriptome repertoire that produces the plasticity of the proteome. Accumulating evidence supports that regulation of alternative transcripts play pivotal functions in eukaryote development, tissue identity^9^, and response to environmental stress^10^. In plants, high-throughput RNA-seq analysis has shown that alternative promotor usage, splicing and polyadenylation in Arabidopsis and other crops^10,11^ are significantly more prevalent than previously expected. Functional studies found that transcription start site (TSS) selection produces truncated proteins which change their subcellular localization^11^, and that regulated alternative splicing controls plant flowering^12^ and affects microRNA biogenesis^13^. Transcription end site (TES) selection and alternative polyadenylation (APA) are also known to regulate plant flowering^14^. Therefore, regulation of RNA transcription and processing significantly affects multiple aspects of plant growth and development.

However, currently available gene models in cotton genomes are mostly derived from computational prediction or assembled only from short reads RNA sequencing data, which are incomplete or even inaccurate. Specifically, the Cottongen^15^ and CGP^6^ gene annotations for the AA genome contain only coding sequences, essentially missing other functional regions, such as 5′UTRs and 3′UTRs (UTR, Untranslated Region). Incompleteness of the cotton transcriptome hinders the investigation of molecular mechanisms for various biological processes.

The next-generation sequencing (NGS)-based RNA-seq which generates millions of short reads, is often used for gene expression profiling; however, the method cannot identify accurate full-length transcripts, and potential amplification biases can be introduced during library construction^16^. Pacbio sequencing offers long reads, with average read lengths over 10 kb, and has been applied to profile the transcriptomes of human^17^, maize^18^, sorghum^19^, and tetraploid cotton (*G. hirsutum*)^20^ with the Iso-seq protocol. However, Pacbio sequencing is hindered by lower throughput, higher error rate (11–15%) and higher cost for identifying transcript isoforms, as in a recent review^21^. The hybrid sequencing, which integrates the strengths of NGS and Pacbio sequencing, has improved AS identification^22^, however, for accurate identification of the 5′ and 3′ ends of transcripts (TSS and TES, respectively), the hybrid sequencing remains inadequate. The most reliable and precise methods for identifying 5′ and 3′ ends can still be best achieved with NGS-based Cap Analysis Gene Expression (CAGE-seq)^23^ and PolyA-seq^24^. The computational method GRIT reconstructed gene models from RNA-seq short reads data by integrating CAGE-seq and PolyA-seq data for the *Drosophila* genome, and has obtained better results than Cufflinks with only RNA-seq data^25^.

To systematically exploit the transcription landscape of *G. arboreum*, a diploid cotton species, here we combine four complementary high-throughput techniques: Pacbio Iso-seq for directly reading full-length isoforms, deep-depth strand-specific RNA-seq (ssRNA-seq) for quantifying expression and splicing, as well as specialized CAGE-seq and PolyA-seq for accurately defining the transcriptional initiation and polyadenylation sites, across 16 tissues including ovules at four developmental stages. We develop an efficient computational pipeline to take full advantage of each technique and generate a high-resolution transcript map. We discover and validate different modes of gene expression regulation in cotton development including alternative promoter usage, splicing hotspot and microexon switch, polycistron, and alternative polyadenylation site selection. The results from this study provide a highly reliable panoramagram of the transcription output in *G. arboreum*, which builds a foundation for studying phenotypic and functional variations in cotton.

## Results

### Multi-strategic RNA-seq for high-resolution RNA landscape

In previous studies, we assembled the *G. arboreum* genome^6,7^. To systematically identify complete and accurate gene models including 5′UTRs and 3′UTRs and capture splicing isoforms for *G. arboreum*, we prepared RNA from 16 tissues, including anther, stigma, petal, bract, sepal and whole flower at 0 days post-anthesis (DPA), phloem, leaf, seedling root, seedling stem, cotyledon, seed, and ovules at four developmental stages (0, 5, 10, and 20 DPA) (Supplementary Fig. 1a). Pacbio long reads sequencing was performed on mixed RNA samples from the 16 tissues reaching close to the saturated depth based on computational sampling analysis (Supplementary Fig. 1b), which yielded 3,332,714 reads of insert (ROI) filtered from 64 SMRT cells, in a total 92.8 Gb size of 5,204,697 raw reads. For each of the 16 samples, we also performed ssRNA-seq in biological duplicates, generating a total of 1,517 million clean reads (455.1 Gb in size). To identify accurate TSSs and TESs, we carried out CAGE-seq (on average 83.1 million clean reads per sample) and PolyA-seq (on average 68.8 million clean reads per sample) for each of the 16 tissues. The statistics of all sequencing libraries is summarized in Supplementary Table 1–3 and Supplementary Data 1.

**Fig. 1:**
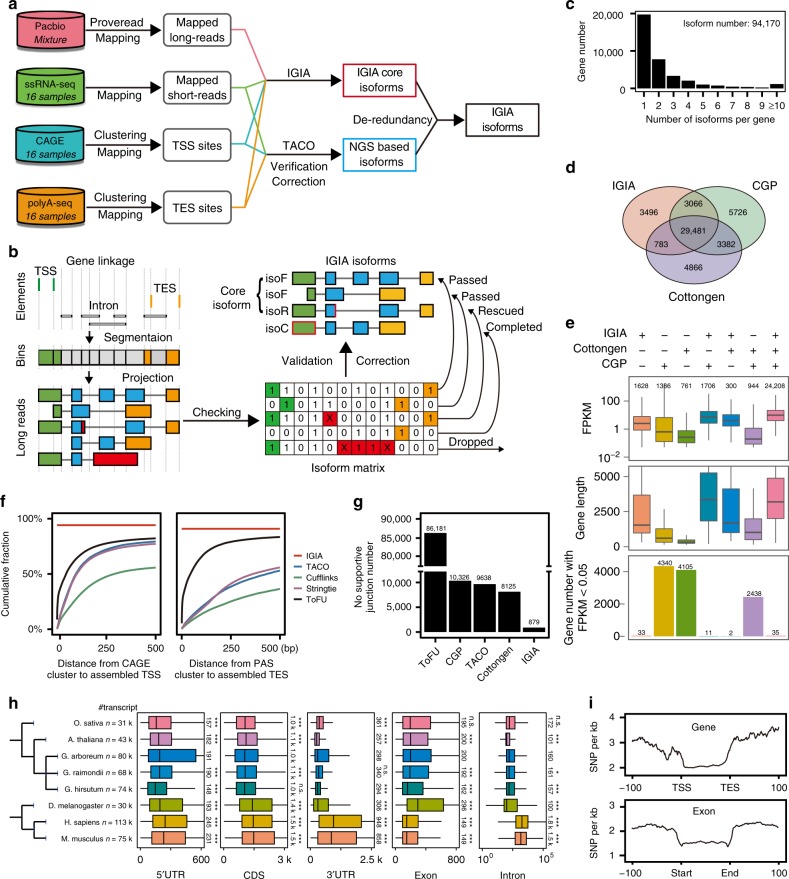
Integrative multi-strategic RNA-seq for the high-resolution RNA landscape in *G. arboreum*. **a** Experimental design and analysis workflow for Integrative Gene Isoform Annotation (IGIA). **b** Schematic illustration of IGIA strategy for identifying accurate isoforms. See details in Methods section. **c** Distribution of the number of isoforms per gene. **d** Venn diagram of gene annotations comparisons between CGP, Cottongen and IGIA. **e** Distribution of FPKM, gene length, and number of lowly expressed genes in subgroups of genes. **f** The deviation between TSS (left, CAGE-seq) and TES (right, PolyA-seq) peaks compared with those assembled from IGIA and other methods. **g** The number of unique splicing junctions only supported by ToFU, CGP, TACO, Cottongen, and IGIA. **h** Length distribution of the 5′UTR, CDS, 3′UTR, exon, and intron, from IGIA annotation for cotton compared with seven other species. The median lengths and significances of difference were marked (*p-value < 0.05; **p-value < 0.01; ***p-value < 0.001). **i** SNP distribution on composite gene body (top) and exon (bottom) of IGIA genes. The source data underlying Fig. 1e are provided as a Source Data file

To take advantage of each of the high-throughput technique, we developed an integrative analysis pipeline to build transcript models. For regions supported with Pacbio long reads, we utilized the developed tool termed IGIA (Integrative Gene Isoform Assembler), while for regions without long reads, we applied customized TACO pipeline recently developed^26^ (Fig. 1a). In the IGIA method, gene elements including TSSs, TESs, and introns, were identified from CAGE-seq, PolyA-seq and ssRNA-seq reads, respectively. Then, the polished long reads were segmented based on the identified elements. Importantly, we validated each long read on every element based on short reads, corrected errors and completed partial isoforms whenever possible, and classified them into different categories of isoforms (Fig. 1b and Supplementary Fig. 1c). Integrating the IGIA core isoforms (IsoF and IsoR) and IsoC, and the isoforms from the adjusted TACO pipeline, we identified a total of 94,170 transcripts from 36,826 genes (Supplementary Data 2, 3). Among IGIA cotton genes, 56.7% have only one transcript, and 17,101 genes have two or more transcripts (Fig. 1c). Our IGIA gene set had a large fraction overlapping with the previous CGP^6^ and Cottongen^15^ annotations, and contained more than 3400 new genes (Fig. 1d). For genes not annotated in IGIA, their expressions were extremely low or their lengths were too short (Fig. 1e).

For assessing the 5′ and 3′ annotations in IGIA, we computed the distances of annotated TSSs and TESs from different pipelines with peaks identified by CAGE-seq and PolyA-seq, respectively (Fig. 1f). Because IGIA utilized the information from CAGE-seq and PolyA-seq, the IGIA gene set had the smallest deviation compared with the peaks. The cumulative fraction curves show that the transcripts from ToFU^27^ annotations with Pacbio long reads have higher resolution for 5′ and 3′ ends than TACO, StringTie and Cufflinks, indicating that short reads sequencing data alone are insufficient for identifying accurate TSSs and TESs, especially TESs.

Next, we assessed the annotated splicing junctions in this study, by comparing with four sets of junction sites (JSs), including predictions from ToFU, TACO, and the CGP and Cottongen annotations (Supplementary Fig. 1d and Supplementary Data 4, 5). Among the JSs, 99.3% (121,912/122,791) of IGIA JSs were also supported from other predictions (Supplementary Fig. 1d), and IGIA had the fewest number of specific JSs (Fig. 1g), indicating less false positives. Notably, both CGP and Cottongen had a considerable number of JS annotations that were different from those deduced with IGIA (Supplementary Fig. 1d). To evaluate these JS differences, 174 pairs of different JSs were randomly selected in the genes with varying expresssion levels (0–25%, 25–50%, 50–75%, and 75–100%). The Sanger sequencing results from the two comparison groups (84 for CGP *vs*. IGIA and 90 for Cottongen *vs*. IGIA, respectively) showed that about 98% of the investigated JSs exactly match with IGIA annotations (Supplementary Fig. 2a, Supplementary Data 6, 7). The representative cases are shown in Supplementary Fig. 2b–f. In addition, the transcript set assembled using ToFU with only Pacbio long reads revealed 86,181 unique JSs which, however, were unsupported by any other method, and the JSs actually contained many boundary errors (74.26%) and bubble errors (20.74%) (Supplementary Fig. 1d right bottom), two common types of errors in Pacbio sequencing (Supplementary Fig. 1e). This, to a large extent, explains the higher number of JSs identified using Pacbio than NGS RNA-seq, a problem also been noted in a previous report^19^. In summary, these evaluations showed the IGIA pipeline discovered more reliable and complete JS annotations than the predictions from ToFU or TACO and previous CGP and Cottongen annotations for the *G. arboreum* genome.

Based on the IGIA gene structure annotations, we characterized and compared multiple genomic features, such as length of UTRs, CDS, exons, and introns in *G. arboreum* and other seven genomes for protein-coding genes (Supplementary Table 4). The overall length features of *G. arboreum* were closer to other plants than animal genomes, as expected. However, the *G. arboreum* genome had unique features including longer 5′UTRs, wider range of 5′ and 3′UTR lengths than *Arabidopsis* and rice, and longer introns than *Arabidopsis*. Interestingly, compared with two other cotton species, the *G. arboreum* genes had wider range of 5′ and 3′UTR lengths (Fig. 1h), although the discrepancy might result from incomplete 5′UTR and 3′UTR annotations in other genomes.

To explore natural sequence variations on these functional elements, we analyzed the distribution of 17,883,108 high-quality single nucleotide polymorphisms (SNPs) from 230 *G. arboreum* lines^7^ in IGIA genes and different categories of elements including 5′UTR, CDS, 3′UTR, and introns. As expected, genic regions possessed significantly fewer SNP mutations than intergenic regions, and the CDS regions had the lowest natural variation frequency (1.65 SNP/kb) (Supplementary Fig. 3a). Notably, sharp transitions of SNP density were observed around TSSs and TES at the gene level, and exon-intron boundary, as well as UTR-CDS boundary at the mRNA level (Fig. 1i and Supplementary Fig. 3b). An obvious valley was observed around 30–40 nt upstream of the TSS (Fig. 1i top), where TATA box motif is enriched. These results reflect the accuracy of IGIA annotation of TSSs, TESs, and splicing JSs.

### Gene expression dynamics across 16 tissues in *G. arboreum*

Based on the improved gene annotation, we next attempted to build a spatio-temporal dynamic transcriptome atlas for *G. arboreum*. We used our ssRNA-seq data to quantify gene expression across 16 tissues including ovules at four developmental stages (0, 5, 10, and 20 DPA) in two biological replicates. The average correlation coefficients between biological replicates for all tissues were greater than 0.95, indicating high quality and reproducibility of our data (Supplementary Fig. 4). We computed the FPKM (number of Fragments Per Kb per Million mapped reads) values for IGIA genes across all tissues (Supplementary Data 8), and investigated their similarity and specificity. Three groups of tissues were discovered based on the expression correlation matrix (Supplementary Fig. 5a): the three reproductive tissues including stigma, anther, and petal; four developmental stages of ovules and seed; other vegetative tissues and the whole flower. These results fit well with our expectation: the ovules at different developmental stages show the most similar gene expression, while vegetative tissues and reproductive tissues differ significantly, thus being grouped separately.

To provide a global view on gene expression across 16 tissues, we made a maximum-value normalized expression heatmap clustered by gene, revealing a considerable number of constitutively expressed genes (Supplementary Fig. 5b). The ubiquitously expressed genes in more than 12 tissues (10,919) accounted for almost 42.46% of expressed genes (Supplementary Fig. 5c). To identify tissue-specific genes, we used an entropy-based metric (see Methods) to quantify the tissue specificity of all genes (Supplementary Fig. 5d). A total of 5702 genes with score larger or equal to 1 were identified as tissue-specific (Supplementary Fig. 5d and Supplementary Data 9). Hierarchical clustering of these genes showed the male reproductive organ (anther) had the largest number of tissue-specific genes. The homologous genes *GhMYB25*, *GhHOX3*, and other factors important for fiber initiation and elongation in *Gossypium hirsutum*^28^, showed an obvious specific expression patterns in ovule tissues (highlighted box in Supplementary Fig. 5d). To further validate the reliability of our data, we randomly selected 16 genes from each tissue-specific group (Supplementary Fig. 5e top) and measured their expression using quantitative polymerase chain reaction (qPCR) across 16 tissues (each with three replicates, 768 reactions in total). The results (Supplementary Fig. 5e bottom) were highly consistent with our ssRNA-seq data. As exemplified by the vacuolar amino acid transporter gene (Supplementary Fig. 5f), the expression signals from ssRNA-seq across 16 tissues clearly revealed an ovule-specific gene, which may have an important function during cotton ovule early development.

To finely resolve the gene expression of fiber at 5, 10, and 20 DPA, the fibers were stripped from the epidermal layer of the ovule for ssRNA-seq (Supplementary Fig. 6a and Supplementary Fig. 4). The expression profiles of six samples were integrated with the above 16 tissue samples for t-SNE plot analysis. The ovule, ovule without fiber (ovule-F), stripped fiber (Fiber), and seed were clustered into one group and clearly separated from other tissues (Supplementary Fig. 6b). Then, all the genes (22,674, FPKM ≥ 1) expressed in the ovule, ovule-F, and fiber, were analyzed to create a heatmap (Supplementary Fig. 6c). Based on the expression trends in fiber from 5 to 20 DPA, the genes were categorized into three groups: Up, Down and Others (Supplementary Data 10). The homologous genes *GhMYB25*, *GhHOX3*, and other factors^28^ associated with fiber development were also included. Most genes belonged to the Up group. Six genes, including putative *GaEX1* (Ga10g01583) and *GaHOX3* (Ga12g00054), were selected for qPCR validation (Supplementary Data 11). The obvious tissue-specific expression of the genes was observed in ovule (0–20 DPA) and fiber (5–20 DPA) (Supplementary Fig. 6d). Due to the fiber separation from ovule, the expression specificity of the genes was more pronounced in pure fiber tissue (Supplementary Fig. 6c, d). In addition, in situ hybridization was used to detect the transcriptional expression of Ga03g00156, which encodes an ATP-dependent DNA helicase or nuclease. Ga03g00156 had strong hybridization signals in the fibers and epidermis of outer integument of ovules as shown in Supplementary Fig. 6e, further confirming its tissue specificity revealed from above RNA-seq data.

This comprehensive and accurate gene expression atlas in *G. arboreum* provides a valuable resource for future studies on cotton tissue specification and development.

### Multiple TSSs and alternative promoter usage

**Fig. 2:**
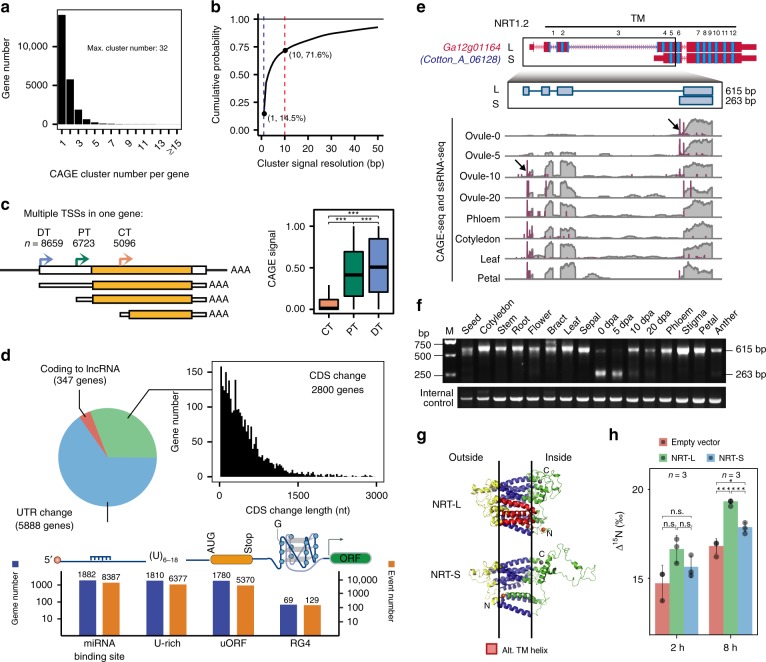
Multiple transcription initiation and alternative promoter usage in *G. arboreum*. **a** Statistics of transcription start site (TSS) per gene. **b** High resolution of TSS identification from CAGE-seq. **c** Statistics of TSS usage signal in genes with multiple TSSs including distal- (DT), proximal- (PT) and coding-TSS (CT). **d** Statistics of 5′ end changes and the distribution of changes in CDS length caused by alternative TSS usage. Potential functional RNA elements in alternative 5′UTR regions including RBP binding, U-rich, RG4, 2nd structure, and uORFs. The occurrences of events and associated genes are shown. **e** Example of a gene with long (L) and short (S) isoforms due to alternative TSS usage (arrows). The sequence encoding the transmembrane (TM) helix domain is marked in blue. CAGE-seq and RNA-seq signals are shown in purple and gray, respectively. **f** 5′ RACE validation of the change in the use of TSS. **g** Predicted 3D protein structures for the long and short isoforms (top: side-view, bottom: top-view). The lost TM helix domains caused by alternative TSS is highlighted in red. **h** The comparison of ^15^NO_3_^−^ uptaking activity between NRT-L, NRT-S, and empty vector cell lines (two-tailed *t*-test, *n* = 3, error bar represents s.d.). The significance levels are indicated by asterisks (*p-value < 0.05; **p-value < 0.01; ***p-value < 0.001), and the median values in box plots are shown. The source data underlying Fig. 2b–d, 2f, and 2h are provided as a Source Data file

CAGE-seq, designed to stringently select for 5′ complete RNA molecules, has been used to identify TSSs at single-base resolution^23^. In the present study, approximately 83 million CAGE-seq reads on average for each of the 16 tissues were successfully mapped to the *G. arboreum* genome, which generated 91,707 TSS clusters using the program paraclu^29^. Requiring an average of TPM (number of Tags Per Million mapped) >0.5 across all tissues, we identified 44,728 TSS clusters, corresponding to 22,863 gene loci (Supplementary Data 12, 13), of which 38.4% had two or more TSS clusters (Fig. 2a).

To estimate the accuracy of the CAGE-seq data, we defined the cluster resolution as the minimum-size interval that accounts for more than 80% of the total signal in the cluster. We assessed the cumulative distribution of CAGE signal across all 16 tissues (Fig. 2b) and found that 14.5% of TSS clusters had single-base resolution and 71.6% of TSS clusters had 10-base resolution. To validate the TSSs, we randomly selected 55 genes to perform the 5′ rapid amplification of cDNA ends (5′RACE) assay in a pooled RNA sample of 16 tissues. The PCR results showed the expected bands in 47 genes (positive ratio = 85.5%) (Supplementary Fig. 7 and Supplementary Data 14). In addition, multiple classical promoter motifs, including Initiator (Inr), TATA box and Y Patch (pyrimidine patch)^30^ in plants, were found enriched near the identified TSSs (Supplementary Fig. 8a). Together, these results reflect the high quality of our CAGE-seq data.

Multiple TSSs in one gene locus would create the potential to rewire the transcription by using an alternative TSS. Next, we examined the genome-wide TSS usage pattern in cotton. Multiple TSSs in one gene were classified into three types: distal TSS (DT), proximal TSS (PT), and TSS in the coding region (CT) (Fig. 2c left). We found that the DT were more likely to be chosen (Supplementary Data 15) and expressed at much higher level than PT and CT (two-tail Wilcoxon rank sum test, *p*-value < 2.2e-16) (Fig. 2c right).

Our data revealed that alternative promoter usage could alter the length of 5′UTR or protein N-terminus in 5888 and 2800 genes, respectively (Fig. 2d top). In alternative 5′UTR regions, we detected enrichment of potential functional RNA elements, including 8387 miRNA binding sites, 6377 U-rich elements, 5,370 upstream open reading frames (uORFs), and 129 RNA G-quadruplexes (RG4), each of which may affect mRNA stability or translational efficiency^31^ (Fig. 2d bottom and Supplementary Data 16). The miRNA binding, U-rich and uORF contributed the most to the identified motifs, indicating these alternative regions of the 5′UTRs might have important regulatory roles in post-transcriptional regulation of gene expression.

Next, we analyzed the consequences on protein for a set of 2800 proteins with alternative TSSs in coding regions. Most of the alternative TSSs, while not causing a frame shift, induced N-terminal truncation with different lengths up to 500 amino acids (Fig. 2d), which would lead to losses of N-terminal subcellular localization signals or protein domains based on predictions (Supplementary Data 16, 17). In *Arabidopsis*, through differential TSS usage, the genes encoding for monodehydro ascorbate reductase (MDAR), stromal ascorbate peroxidase (sAPX)^32^, and glycerate kinase (GLYK)^11^, have been shown to produce both full-length and truncated proteins to target different subcellular compartments. Based on our data, their orthologs in *G. arboreum* also possessed dual promoters and exhibited differential TSS usage among tissues, indicating the genes may be subjected to similar transcriptional regulation for subcellular localization in cotton (Supplementary Data 17).

Further, we analyzed the TSS usage dynamics across 16 tissues and uncovered 6,207 genes with promoter switches between tissues. Interestingly, we found that TSS selection might control the existence or loss of structure domain(s) for genes involved in ABA signaling (protein phosphatase PP2C, Ga06g00624), auxin transport (ABC transporter, Ga14g01845), nitrate transport (NRT1.2, Ga12g01164), and membrane signal transduction (leucine-rich repeat receptor-like protein kinase, Ga11g00886) (Supplementary Data 16). Figure 2e shows a representative example of our data, Ga12g01164, a homolog of *AtNRT1.2* encoding nitrate transporter. Our ssRNA-seq and CAGE-seq are consistent with the possibility that ovules at 0 and 5 DPA mainly utilize CT TSS to express a short mRNA *NRT-S*, whereas late stage ovules (10 and 20 DPA) and other tissues switch to DT TSS to express a longer mRNA *NRT-L* (Fig. 2e).

Indeed, our 5′RACE assay results confirmed the TSS switch (Fig. 2f). The NRT1.2 has 12 trans-membrane (TM) domains, but due to the change from distal to proximal TSS, *NRT-S* encodes an N-terminal truncated version with loss of the four TM domains, which might induce a protein conformational change from the compact to relaxed state for the TM cluster based on 3D structure modeling (Fig. 2g). For comparing their activity on NO_3_^-^transport, we performed ectopic expression for NRT-L and NRT-S in HEK-293 cells to test the isotope ^15^NO_3_^-^ uptake. The results from the assays at 2 and 8 h further confirmed their difference in the activity of NO_3_^-^ uptake, for which NRT-S was significantly lower than NRT-L (Fig. 2h). These results imply that at early developmental stage (0 and 5 DPA), the ovule might have unique nitrogen transport and metabolism, distinct from other tissues.

The above results indicate that differentially regulated alternative TSSs are a common feature in cotton mRNAs, which usually generate alternative N-termini in mRNA or protein to regulate tissue specification and development.

### Developmentally regulated TES selection

**Fig. 3:**
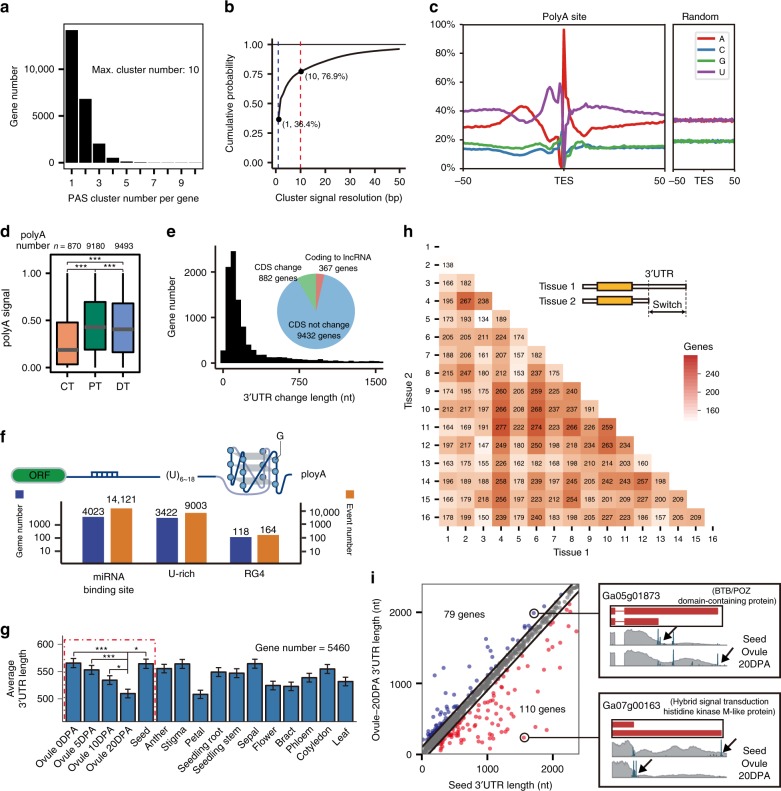
Multiple transcription termination and alternative 3′UTR usage in *G. arboreum*. **a** Statistics of transcription end site (TES) per gene. **b** Cumulative curve of base resolution of the TES cluster signal from PolyA-seq. **c** The nucleotide composition around the TES. **d** Statistics of TES usage in genes with multiple TESs including distal- (DT), proximal- (PT) and coding-TESs (CT). **e** Statistics of effect on RNA (pie chart) and the distribution of change of CDS length due to the use of an alternative TES. **f** Potential functional RNA elements in the alternative 3′UTRs including RBP binding, U-rich, and RG4 motifs. **g** Average 3′UTR length across tissues (error bar represents s.e.m.). **h** Number of genes with alternative TES usage between pairwise tissues. **i** Genes with changes in APA between ovule (20 DPA) and seed (left), and two representative gene examples (right). The significance levels are indicated by asterisks (*p-value < 0.05; **p-value < 0.01; ***p-value < 0.001), and the median values in box plots are shown. The source data underlying Fig. 3b–h are provided as a Source Data file

Alternative TES selection or APA generates mRNAs with various 3′ ends that differ either in coding sequence or in 3′UTRs, which contributes to the transcriptome complexity by regulating mRNA stability, localization, and translation efficiency^33^. In cotton, APA has not yet been systematically investigated. Based on the 3′ ends information from PolyA-seq, we profiled genome-wide TESs for all expressed genes in 16 tissues. From a total of 1.1 × 10^9^ PolyA-seq reads mapped to the cotton genome, we generated 70,502 TES clusters, of which 43,237 TES clusters had an average TPM > 0.5, corresponding to 23,736 gene loci (Supplementary Data 18, 19). Among all expressed genes in cotton, 40.2% had at least two TESs (Fig. 3a), on average, 1.56 polyA sites per gene, less than the 74.9% in *Arabidopsis*^34^ but close to the 47.9% in rice^35^. Similar to the TSS analysis mentioned above, we next assessed the cumulative distribution of polyA site resolution across all 16 tissues, showing that 36.4% of TES clusters had single-base resolution and 76.9% of TES clusters had 10-base resolution (Fig. 3b).

To further confirm the reliability of deduced TESs, we scanned the canonical 3′ end processing motifs, including CFlm, U-rich, and PAS^36^, all which were indeed enriched around the TESs as expected (Supplementary Fig. 8b). In addition, the nucleotide (nt) composition of sequences ±50 nt around the TES was analyzed, and a typical A/U enrichment pattern was found at the PolyA site, similar to previously reported results in *Arabidopsis*^37^ and rice^35^, but not in control sequences (Fig. 3c). These results indicate our TES annotation is reliable and of high resolution.

To examine the APA dynamics across tissues in cotton, we quantified the TES usage from our PolyA-seq data and identified 10,681 genes exhibiting APA (Supplementary Data 18). To differentiate the pattern of TES usage, we classified multiple TESs in single genes into three types: distal TES (DT), proximal TES (PT), and TES in the coding region (CT). Unlike the situation in transcription initiation, the distal TESs (DT) tended to be used less frequently (Supplementary Data 20) than PT, however, DT and PT were expressed at much higher levels than the CT TESs (Fig. 3d). This is highly reminiscent of previous studies in rice^35^, but different with that in mice^24^, in which distal TESs appeared to be used more frequently and express at a higher level than PT. This intriguing phenomenon indicates different preference in plants and animals for the use of alternative TESs.

While most of the 3′ end variations (9432 of 10,681, 87.5%) were limited to 3′UTR, only 8.3% of variations occurred between coding regions and the 3′UTRs; 3.4% would change coding RNA to lncRNA by breaking the open reading frame (ORF) (Fig. 3e). To further dissect the effect of varying 3′UTR lengths, we analyzed the composition of alternative 3′UTR regions and found an enrichment of RNA functional motifs (Fig. 3f and Supplementary Data 21), including miRNA binding site, U-rich elements, and RG4.

Next, we examined shortening or lengthening patterns of the 3′UTRs across 16 tissues, and found that the average length of 3′UTRs showed great variations among different tissues (Fig. 3g and Supplementary Data 22). In particular, gradual shortening was observed during ovule development from 0 DPA to 20 DPA, which then exhibited a sudden reversal to lengthening of the 3′UTR at the final ovule developmental stage, mature seed (dashed box in Fig. 3g). This reproductive development associated variation in 3′UTR length has been reported in mouse spermatogenesis with 3′UTRs being the shortest in spermatids^38^, and in *C. elegans* germline with a bias toward proximal TES in males^39^. The gradual shortening followed by lengthening transition in cotton may be of interest for future studies.

To gain insight into the APA differences from our genome-wide data, we identified the genes with 3′UTR switching between pairwise tissues, as shown in the heatmap (Fig. 3h). The anther, stigma and petal (tissue ID 1–3) showed relative low number of APA switching events compared with other tissues, however, seed and ovules (tissue ID 4–8) exhibited larger numbers of switching cases compared with vegetative tissues (ID 9–11,14–16). Most APA switching cases involved in ovule and seed, implying APA in cotton may play an important role in fiber development and seed maturation.

Among the 1,899 mRNAs expressed in both ovule (at 20 DPA) and seed, 189 genes exhibited APA switching with robust differences (Fig. 3i). In these cases, more genes used the distal polyA sites in seed, such as Ga07g00163, encoding histidine kinase, which might play a role in signal transduction across the cellular membrane^40^. Conversely, some genes, including Ga05g01873, encoding a BTB domain containing protein potentially modulating chromatin structures^41^, tended to use the proximal polyA sites in seed.

Collectively, based on the results, a high-resolution map of PolyA sites in cotton was built, and their sequence features and dynamic regulation during development and tissue specification were revealed.

### Dynamic splicing switch and microexons in cotton

**Fig. 4:**
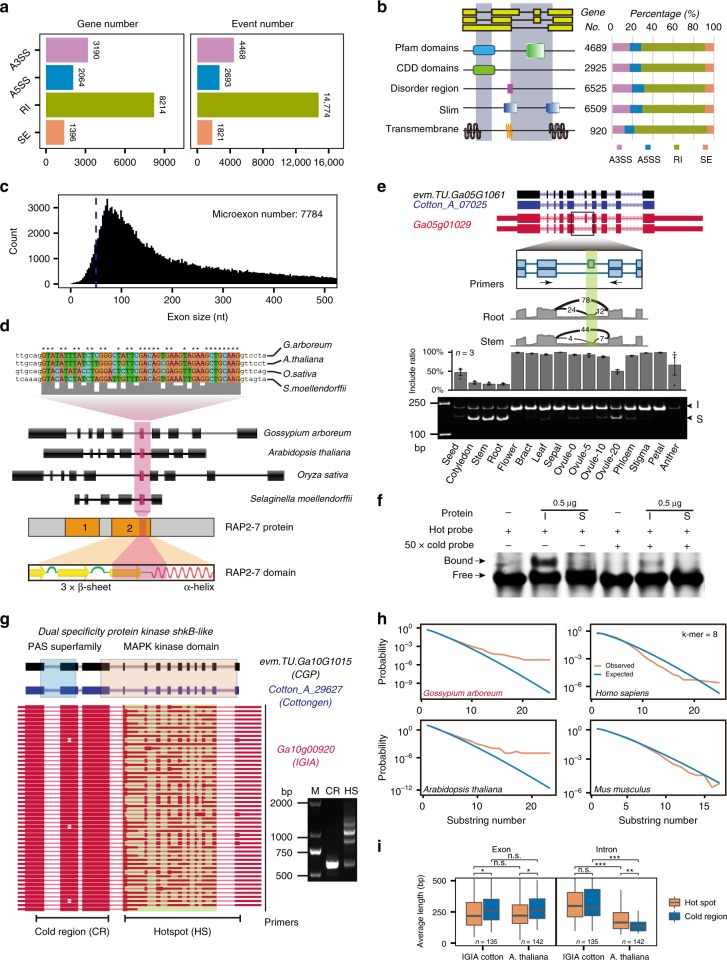
Alternative splicing regulation and hotspots in *G. arboreum*. **a** Statistics of four categories of AS events and associated genes identified in 16 tissues using IGIA annotation. A3SS, alternative 3′ splice site; A5SS, alternative 5′ splice site; RI, intron retention; SE, exon skipping. **b** Effects of AS events on protein functional domains. **c** Size distribution of internal exons. Dashed line: 51 nt threshold. **d** A microexon (ME) located within the AP2 DNA binding domain and conserved in plants. **e** ME validation using RT-PCR. I, inclusion; S, skipping. The bar chart reflects the exon inclusion ratio across 16 tissues (three replicates, error bar represents s.d.). **f** EMSA assay showing two isoforms w/o ME, I, and S, have different DNA binding affinities. **g** Representative gene example with AS hotspot (highlighted in light green color) and RT-PCR validation for the splicing products from the “cold” region (CR) and hotspot (HS). **h** Simulation of AS hotspots in representative plant and animal genomes. **i** Length comparison of exons and introns from HSs and CRs. The significance levels are indicated by asterisks (*p-value < 0.05; **p-value < 0.01; ***p-value < 0.001). The source data underlying Fig. 4e–h are provided as a Source Data file

Alternative splicing (AS) in cotton has been investigated in *G. raimondii* (DD genome)^42^ and *G. hirsutum* (AADD genome)^20^, but not yet in the *G. arboreum* AA genome. Based on the IGIA annotation, we performed a systematic analysis of AS in 23,451 multi-exon genes of *G. arboreum*. In total, we identified 23,756 AS events in 42.1% multi-exon genes (Supplementary Data 23–26), ~2.4 AS events on average in genes with AS (Fig. 4a).

Our results revealed that intron retention (RI) constituted the majority (62.2%) of all AS events in *G. arboreum*, which is the highest among all reported plants. This result has also been found in two other *Gossypium* species. The percentages of three other patterns of AS events were 18.8%, 11.3%, and 7.7% for alternative 3′ splice site (A3SS), alternative 5′ splice site (A5SS), and exon skipping (SE), respectively (Fig. 4a). We further characterized the impact of AS events on protein-coding sequences, especially various functional domains, and found that AS in cotton greatly affects the integrity of protein domains in Pfam and CDD, and sequences with disordered regions, short linear motifs (Slim), and trans-membrane (TM) features (Fig. 4b and Supplementary Data 27–30). Genes with different AS events were randomly selected to test the accuracy of the AS annotation using RT-PCR (Supplementary Fig. 9a-d and Supplementary Data 31). The highly dynamic splicing switches across different tissues from ssRNA-seq were consistent with our RT-PCR findings. The AS quantification for all the IGIA genes based on our ssRNA-seq data shows comprehensive AS dynamic profiles across tissues in *G. arboreum* (Supplementary Data 23–26).

Next, two special types of transcription coupling events were investigated, intronic TSS and intronic TES switching, linking AS and alternative promoters or polyadenylation, respectively^24^. The intronic TSS (or TES) in the skipped terminal exon (Is event) or in composite terminal exon (Ic event) will produce or eliminate a splicing junction, respectively (Supplementary Fig. 10a). Genome-wide survey showed the coupling events between transcription initiation (or polyadenylation) and splicing occurred extensively in cotton, and the Ic event was more widely present in the transcriptome at either 5′ end or 3′ end than the Is event (Supplementary Fig. 10b). Several gene loci of the cotton genome contained both Ic and Is events. Two gene examples with mixed types of events at the 5′ end and 3′ end are presented in Supplementary Fig. 10c, d, showing the gene complexity contributed by intronic TSS or TES.

Microexons (MEs) are a particular class of exons, and their lengths are usually as short as 3 nt^43^. Here we comprehensively characterized MEs in cotton *G. arboreum*. Similar to humans^43^, the length distribution of internal exons showed a sharp decrease around 50 nt (Fig. 4c), and we defined the length threshold for MEs as 51 nt in cotton as previously reported. By scanning internal exons equal or less than 51 nt, 7784 MEs were identified from IGIA transcripts (Supplementary Data 32). Figure 4d shows a 45 nt ME in Ga05g01029 encoding the ethylene-responsive transcription factor RAP2-7 protein, an AP2 domain-containing transcription factor. This ME encodes part of the β-sheet and α-helix of the AP2 domain. Comparing the gene structures of the orthologs from gymnosperm *selaginella moellendorffii* to rice and *Arabidopsis*, the sequence and length of this ME were highly conserved, although the length of other exons in this gene varied greatly among different plant genomes (Fig. 4d). The RT-PCR assays showed the AS of this ME had an obvious tissue-specific patterns in cotton, as shown by the exon inclusion ratios across tissues (Fig. 4e). Furthermore, we examined the DNA binding activity of the RAP2-7 protein with ME skipping (S) or inclusion (I) using an EMSA assay. Figure 4f showed that at the same protein concentration, AP2-I could bind the DNA probe while AP2-S almost failed to bind, indicating the transcription factor RAP2-7 might vary its binding activity to the genome in different tissues via regulated splicing of a microexon, a common mechanism shared by animals and plants.

In addition, several regions in certain genes showed highly enriched AS events, which we termed as AS hotspots (Fig. 4g, highlighted light green box). Based on a statistical model of AS hotspots, we scanned the cotton genome with a high stringency criterion, and detected 135 genes that contained at least one AS hotspot (Supplementary Fig. 10e–g). Gene ontology (GO) analysis of the genes showed the molecular functions were mainly enriched in ATP and RNA binding proteins and protein kinases (Supplementary Fig. 10h). Further analysis on the protein features of AS hotspots indicated that most hotspots affected a conserved protein domain (Supplementary Data 33–35). For example, in protein kinase shkB-like, the AS events tended to concentrate in a hotspot encoding the MAPK kinase domain. The different frequencies of AS in the “cold” region and hotspot for this gene were further confirmed using RT-PCR (Fig. 4g, right). Applying the same computational model, we found AS hotspot also existed in other plants, such as rice and *Arabidopsis*, but not in humans and mice (Fig. 4h). This indicates the AS hotspot might be a plant-specific splicing phenomenon, probably due to plant-specific *cis*-elements and *trans*-factors. Intriguingly, comparison of gene structures between hotspots and cold regions showed the AS hotspots tended to have shorter exons than cold regions, but not the same for introns (Fig. 4i).

In summary, our analysis provides comprehensive information on the alternative splicing landscape in *G. arboreum*, which will be of great value in addressing the regulation mechanism and function of alternative splicing in cotton.

### Discovery of polycistrons and their genomic features

**Fig. 5:**
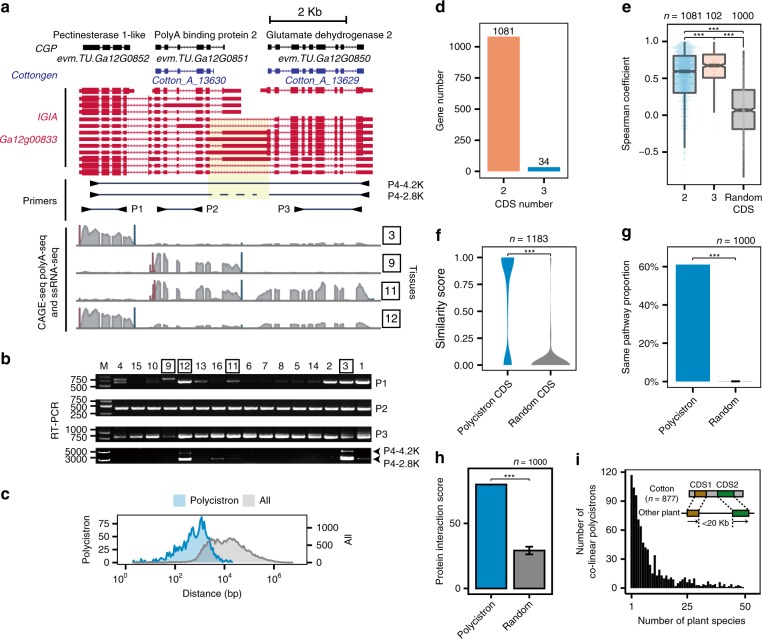
Identification and genomic features of the polycistrons in *G. arboreum*. **a** An example of polycistron with three genes supported by the Pacbio long reads. The genes in the polycistron could be independently transcribed and have independent TSSs and TESs. The probes for RT-PCR validation of the polycistron expression are marked below IGIA transcripts. **b** RT-PCR validation of polycistron transcripts from transcriptional read-through. Four pairs of probes are indicated. **c** Statistics of the distances between CDS pairs in a polycistron compared with the distances between a CDS and its closest CDS in IGIA genes. **d** The number of polycistrons with two and three CDSs identified using IGIA genes. **e** Expression correlation between CDS pairs in a polycistron and random CDS pairs. **f**–**h** Comparison of GO similarity (**f**), proportion in the same KEGG pathway (**g**), and potential protein–protein interactions (**h**) between CDS pairs in a polycistron and random CDS pairs. **i** Co-linear analysis of cotton polycistrons in 54 plant genomes. The significance levels are indicated by asterisks (*p-value < 0.05; **p-value < 0.01; ***p-value < 0.001), and error bar represents s.d. value. The source data underlying Fig. 5b and 5f–i are provided as a Source Data file

Polycistronic transcription, the co-transcription of more than one open reading frame (ORF) or gene from one single promoter to make a polycistronic mRNA, is pervasive in prokaryotes and fungi^27^, but rare in eukaryotes. In plants, a rare example was observed in tomato, a bicistronic unit for glutamyl kinase and glutamyl phosphate reductase loci, which resembled prokaryotic polycistronic operons^44^. Analysis of our IGIA annotation for full-length transcripts in *G. arboreum* indicated many adjacent loci in the cotton genome exhibited polycistronic transcription spanning two or more ORFs, which were annotated as independent monocistronic genes in Cottongen and CGP. Figure 5a and Supplementary Fig. 11 show a representative tricistron and bicistron, respectively (Supplementary Data 36).

In Fig. 5a, the tricistron spans three genes, *Pectinesterase 1-like*, *PolyA binding protein 2*, and *Glutamate dehydrogenase 2* three genes, and the bicistron spans the former two genes. Results from RT-PCR and Sanger sequencing showed the polycistronic transcripts did have two isoforms with lengths of 4.2 and 2.8 kb, due to an alternative intron retention event. The TSS and TES signals from mixed tissues indicated the three genes might have their own transcription units and exhibit different expression patterns with the polycistron, which were supported by our ssRNA-seq and RT-PCR validation (Fig. 5b). The tissue-specific expression of the polycistron implied that it may be activated under the control of tissue specification signals. Based on a stringent criterion, 1115 polycistrons (1081 bi- and 34 tri- cistronic transcripts) in *G. arboreum* were identified (Fig. 5c and Supplementary Data 37), corresponding to 5% of all annotated loci in this study.

To understand potential mechanisms and functions of polycistrons, we investigated several genomic features in these polycistrons and control genes. The distances between ORFs inside the polycistron (between the stop codon of the first ORF and the start codon of the second ORF) were significantly shorter than between monocistronic genes (Fig. 5d). Genes from the same polycistron showed stronger co-expression correlation across tissues than random controls (Fig. 5e). In addition, genes in the same polycistronic transcript tended to have similar GO-terms, locate in the same KEGG pathways, and interact with each other (Fig. 5f–h, respectively), probably due to better coordination through their co-transcription. To investigate the origin of polycistron, we performed co-linear analysis for the polycistrons with other plant genomes. The results (Fig. 5i) indicated that many polycistrons have co-linearity with the gene loci in a large number of plants. The co-linearity of some polycistrons exist in more than 40 species, the conservation of which indicates polycistrons may play important regulatory roles.

In a recent *Arabidopsis* study, under the salt stress or in CTD PHOSPHATASE-LIKE 4 knockdown lines, transcriptional read-through from a small nuclear RNA to its downstream gene was activated to produce a polycistron^45^. Similarly, read-through was considered to be wasteful or harmful transcription of intergenic sequences, which may interfere with transcription of downstream genes^46^. Our results provide evidence supporting polycistron or transcriptional read-through is a widespread phenomenon in cotton growth and development, indicating that polycistronic transcription is not due to transcriptional abnormality, but instead a gene expression regulatory strategy used in plants.

## Discussion

We have compiled a landscape of genes for cotton *G. arboreum* with accurate TSSs, TESs, splicing sites, and complete collection of isoforms, as well as their dynamic profiles across multiple tissues. This comprehensive and systematic transcriptome study integrated multiple RNA-seq techniques and will provide an important reference for cotton and plant researchers.

The emerging new RNA sequencing technologies require a continuous update of analysis tools to further improve the annotation accuracy of the transcriptome. Inspired by the GRIT algorithm^25^, IGIA integrated CAGE-seq and PolyA-seq to determine the gene start and end boundaries, respectively. For gene body regions, GRIT only used short reads data from RNA-seq to algorithmically reconstruct isoform structures, which was challenging^16^, while IGIA took the full-length structures from Pacbio long read as isoform skeleton, and used short reads from RNA-seq to correct local errors in the long reads, thus avoiding the error-prone reconstruction only from short reads. Integrating the four modalities of RNA-seq data, IGIA showed good performance on gene annotation supported by various experimental validations in this study. In this study, IGIA tends to detect isoforms with specific differences on TSSs or TESs (Supplementary Fig. 12a, b), and with reliable expression signals (Supplementary Fig. 12c, d). Requiring multiple evidences on splicing junctions, IGIA aims to identify reliable gene structures, accompanied by potential underestimation of isoforms. IGIA would be further optimized to reduce computation time and memory requirement, and to improve the error-correction capability on long reads to enhance its sensitivity.

In previous genome-wide association studies (GWAS), many agronomic trait-associated sites in cotton have been identified, however, non-CDS polymorphisms are difficult to interpret or prioritize for an in-depth study. With our complete catalogues of functional elements, including newly available 5′ UTRs and 3′ UTRs, researchers can make novel hypotheses for further dissection of important sequence variants outside the CDS. In our investigation, we integrated 932 GWAS sites on the AA genome of *G. arboreum* (Supplementary Data 38) and observed that many GWAS sites were localized in regulatory regions including transcription initiation and termination sites, alternative 5′/3′ UTRs, and constitutive or alternative splicing (AS) sites (Supplementary Fig. 3c and Supplementary Table 5). This provides an important foundation for further decoding the molecular mechanism from these GWAS sites to the important agronomic traits, such as fiber length and quality in cotton.

Aberrant use of one promoter over another in humans has been reported to cause various diseases, including cancer^47^. However, in plants, there has been limited reports on identifying precise TSSs, only in maize^48^ and *Arabidopsis*^11,30^. In the present study, we have identified 91,707 TSS clusters for 26,763 gene loci, almost 40% of which had multiple promoters, indicating extensive usage of alternative promoters in the cotton genome. To date, the functional significance of alternative promoters and their roles in plant development have largely been unexplored, with the rare exception of the well-characterized *Arabidopsis* gene, *GLYK*^11^ and *BES1*^49^. In this study, 2800 genes with different protein isoforms (CDS change) caused by alternative TSS selection in cotton *G. arboreum* (Fig. 2d) were identified, including the genes whose *Arabidopsis* homologs were reported to target different subcellular localization by use of an alternative promoter. Moreover, the effects of TSS selection on protein length might not only be limited to the subcellular targeting signal sequence in N-termini, but also change protein structure and function (e.g. loss of TM structure in nitrate transporter, NRT1.2, which might attenuate the NO_3_^-^ uptake activity.) (Fig. 2f–h). These results broaden the understanding of plant TSS selection, thus providing new research perspectives for plant scientists.

The *cis*-elements, which are commonly present in UTRs of mRNAs, can regulate the RNA stability, translation efficiency, transport, and subcellular localization of the translated proteins^50^. In this study, 5888 and 9432 genes were identified in which the selection of TSS and TES could produce distinct 5′UTRs and 3′UTRs, respectively. Both of the variable regions enrich different functional elements. U-rich motifs have been reported to act as control signal to regulate 3′-end processing^36^, and as *cis*-elements to target mRNA to cytoplasmic processing-body during ethylene signaling in *Arabidopsis*^51^. In the present study, the U-rich motif was found widely present in the 5′UTR and 3′UTR, indicating the motif might have an important role in post-transcriptional regulation.

In addition, results from the present study revealed uORFs frequently occur in the variable regions of the 5′UTR due to use of an alternative TSS. uORFs often function as translation repressors of their downstream major ORFs^52^. In a recent study, a TSS switching to shortened 5′UTR caused skipping of an uORF, evading the uORF-mediated translational inhibition and/or mRNA decay^53^. Our analysis showed 19.6% of genes in *G. arboreum* contained small stretches of uORFs in the 5′UTR of mRNAs, of which 25.1% (1780) were located in alternative 5′UTRs, indicating TSS selection-controlled uORF translational repression could be a pervasive regulation mode in plants.

Recently, Wang et al. performed Pacbio-seq and studies alternative splicing and polyadenylation in an allotetraploid cotton (*G. barbadense*)^20^. The authors found ~4.8 AS events and 2.82 polyA sites per gene, which are larger than those numbers (2.4 and 1.56) in *G. arboreum*, probably due to the conservativeness of IGIA in identifying isoforms or greater complexity of RNA processing in tetraploid cotton than diploid cotton. In this study, important phenomena, including AS hotspot and polycistronic transcription, were discovered. The AS hotspot-containing genes tended to encode proteins with functions enriched in ATP binding, RNA binding, and receptor protein kinase (Supplementary Fig. 10h), and AS hotspots tended to be located in the conserved protein domains including RNA recognition motifs (such as RRM and PPR), protein interaction domains (such as WD40 and BTB/POD), and protein kinase activation regions. For example, in the RNA binding protein, the high variability in the AS hotspot region may increase the plasticity for expanding the RNA recognition range or fine-tuning its affinity. However, the biogenesis and functions of AS hotspots and polycistrons in plants are both issues that warrant further investigations.

## Methods

### Plant materials

The diploid AA genome cotton *G. arboreum* cv. Shixiya1 (SXY1) was grown in soil mixture in a fully automated greenhouse, which mimicked the natural environment for cotton growth. For RNA sequencing, the anther, stigma, petal, bract, sepal and whole flower were collected at 0 DPA. The phloem and leaf tissues were collected at the flowering stage. The root, stem and cotyledon were collected from seedling plants when growing out two fully expanded leaves. The four ovule samples were respectively collected from the ovary of flowers at four developmental stages (0, 5, 10, and 20 DPA). The samples of fiber and ovule (without fiber) were manually separated from ovules at corresponding development stages. The dry seeds were collected from matured cotton bolls. All tissues were immediately frozen in liquid nitrogen and stored at -80 °C until RNA extraction.

### Library construction and sequencing

Total RNA was isolated using a modified protocol as described^54^. For Pacbio sequencing, the RNA samples from 16 tissues were pooled to construct the cDNA library using the SMARTer PCR cDNA synthesis kit according to the manufacturer’s instructions. Next, the cDNA library was size-fractioned to 0.5–1, 1–2, 2–3 Kb, and >3 Kb and single-molecule sequencing on Pacbio RS II was performed using P6-C4 kit with 240 min of movie time for each flow cell, respectively (Nextomics Biosciences Institute, Wuhan, China). The ssRNA-seq, CAGE-seq, and PolyA-seq for 16 tissues of RNA samples were performed on Illumina Hiseq 3000 platform with PE150 mode. For ssRNA-seq, the 16 tissues of total RNA samples with two biological replicates were first depleted of rRNAs with Ribo-Zero rRNA Removal Kit (Epicentre, Madison, WI, USA), and then the strand-specific cDNA libraries were prepared according to a published protocol^55^, and finally constructed into sequencing libraries using NEBNext UltraTM Directional RNA Library Prep Kit (New England Biolabs, USA) for Illumina. The CAGE-seq and PolyA-seq libraries for each tissue were constructed according to RAMPAGE^23^ and 3′READS+^56^ protocols, respectively. For removing PCR duplication in CAGE-seq and PolyA-seq, the adapter primers were modified and random 6xN barcodes were inserted.

### Data processing

For ssRNA-seq, the raw reads were filtered to remove the adaptors and low-quality bases using Trimmomatic (v0.36). Filtered reads were first aligned to the cotton genome^7^ using STAR (v2.5.3a) in end-to-end mode to scan splice junctions. Then the reads were realigned to the genome with splice junctions from the 16 tissues. The FPKM values and counts in different genomic features were calculated using StringTie (v1.3.3) and subread (v1.5.3), respectively.

For Pacbio data, due to the high rates of indels and mismatches, the long subreads were corrected with ssRNA-seq data by program proovread. The corrected reads were then aligned to the genome using the STARlong program with the following parameters: --outSAMattributes NH HI NM MD --readNameSeparator space --outFilterMultimapScoreRange 1 --outFilterMismatchNmax 2000 --alignTranscriptsPerWindowNmax 10000 --scoreGapNoncan -20 --scoreGapGCAG -4 --scoreGapATAC -8 --scoreDelOpen -1 --scoreDelBase -1 --scoreInsOpen -1 --scoreInsBase -1 --alignEndsType Local --seedSearchStartLmax 50 --seedPerReadNmax 100000 --seedPerWindowNmax 1000 --alignTranscriptsPerReadNmax 100000.

For CAGE-seq data, the adapters in raw reads were removed using Cutadapt (v1.13). The 5′ random barcode in Read 1 and GGGG close to the barcode were trimmed, and only the reads with insert sequence length longer than 75 nt were used. Then, the potential rRNAs and PCR duplicated reads were removed according to the signature of barcode and the mapped genomic position from STAR in end-to-end mode. Reads with a mapping quality score (MAPQ) ≥20 were used for further analysis.

For PolyA-seq, the adapters in raw reads were removed using Trimmomatic. The 5′ random barcode in Read1 and Read2, polyA stretch in Read1 and polyT stretch in Read2 were trimmed. Only the reads with insert sequence length ≥50 nt and polyT length ranging from 11 to 15 nt were used. The potential rRNAs and duplicated reads from PCR were removed based on the signature of barcode and the mapped genomic position using STAR in end-to-end mode. Reads with a MAPQ ≥20 were used for downstream analysis.

The TSS and TES reads, from CAGE-seq and PolyA-seq respectively, were clustered using the program paraclu^29^ (parameters: minValue 20, -l 200, -d 5). The clusters across different tissues were merged. Potential TSS and TES clusters with TPM ≥0.5 in any of the 16 tissues and with the largest signal ≥10 in at least three tissues were considered as reliable TSS and TES clusters. The summit having the largest signal in a cluster was considered a TSS or TES position, which was used for reconstructing IGIA transcripts.

### IGIA pipeline

For potential gene locus from linkage analysis, individual elements including TSS, exon, intron, and TES, were first identified from all the sequencing data. To simplify the gene models and speed up construction of isoforms for multiple TSS clusters closer to each other, 400 nt cutoff was chosen to merge clusters based on the statistical analysis about the relationship curve (where second-order difference close to zero) between the remaining cluster number with varying distance cutoffs (Supplementary Fig. 12a). The position with the highest signal in the merged cluster was chosen as the TSS candidate for downstream analysis. The same cutoff was chosen for TES cluster based on the statistical analysis in Supplementary Fig. 12b.

Isoform matrix was built by projecting Pacbio full-length reads into segmented Bins (0 for intron, 1 for exon). Each row, a potential isoform, from the isoform matrix was examined for the evidences of splicing junctions, TSS, and TES. Fully supported isoforms were classified as isoF, partial isoforms were completed as isoC, and isoforms with conflicts to those evidences were rescued with faithful elements as isoR if possible, or otherwise discarded.

In the IGIA pipeline, each transcript was defined as the individual path of genomic region split by TSSs, TESs, and splice junctions. First, the entire genome was scanned with 100 bp window and divided into linkage groups with ssRNA-seq and/or Pacbio Iso-seq signal. For each linkage group, ssRNA-seq and Pacbio reads were used to identify credible junctions with sufficient reads support. Then, the TSSs, TESs, and validated splice junctions were mapped onto genome to divide the genome into indivisible segments. The region containing TSS or TES was termed the “TSS or TES segment”. Next, Pacbio reads were mapped into segments and validated, filtered and corrected to obtain the isoform path using the junction evidences.

For each Pacbio isoform path, the corresponding transcript was termed a full-length isoform (isoF) if it started with TSS segment, ended with TES segment, and all splice junctions were supported. If the isoform path missed at least one of the TSS or TES segment, all splice junctions were supported, and it could be completed using the isoF path that matched its boundary segment, then the corresponding transcript was termed a complete isoform (isoC). For reads that could not be completed by full-length isoform, they were merged with complementary reads as merged isoform (isoM), if there was no conflict in their overlap region. If at least one junction in this isoform path could not be supported by the data, this error was corrected in conjunction with the isoF path. The corresponding transcript was termed a rescued isoform (isoR) if it could be corrected. For a long read with unsupported junctions not rescued by the above methods, we removed those wrong junctions and filled the gaps by enumerating exons at the loci identified from NGS RNA-seq; the resulted isoform was termed partial isoform (isoP).

### TACO pipeline

For these genes without Pacbio long read support, revised TACO assembly pipeline was used to reconstruct transcripts from ssRNA-seq reads. First, TACO was performed to obtain the original version of gene annotation^26^. Based on our analysis for expression (FPKM) of single-exon and multi-exon fragments identified in TACO (Supplementary Fig. 12c,d), FPKM = 0.5 was chosen as the cutoff to filter approximately 50% of low expressed single-exon fragments (Supplementary Fig. 12c). Moreover, because multi-exon fragments have longer length and higher average expression, FPKM = 0.2 was chosen as the cutoff to filter approximately 5% of multi-exon fragments (Supplementary Fig. 12d). Thus, the lowly expressed fragments or noise signals were excluded for further analysis. If there was a TSS or TES cluster within 500 bp from a transcript boundary, the boundary was fixed by this cluster. The transcripts from this customized TACO pipeline were termed as NGS-based isoform (isoN).

### IGIA and TACO integration

Two methods described above were to construct the IGIA gene assembly. For regions with Pacbio support, the gene assembly was predicted using the IGIA pipeline. To obtain a complete cotton gene annotation, the gene locus without Pacbio coverage was supplemented with TACO assembly and modified TSSs and TESs. IsoF and isoR were treated as the core IGIA annotation due to their highest reliability. The IGIA and TACO transcripts were collectively referred to as the complete IGIA annotation. Nearly 50% of the genes <1 kb were not covered by Pacbio reads, which accounted for 58% of the genes not hit by Pacbio. For genes 1–3 and >3 kb in length, Pacbio reads covered 85% and 95% of genes, respectively.

For each IGIA isoform, coding probability was predicted using cpat (v1.2.2). The isoform with coding probability ≥50% was classified as coding isoform, and the longest ORF predicted using ORFfinder (v0.4.3) of NCBI was utilized as the CDS region. Gene cluster was built using the UCSC Genome Browser tools.

### Functional annotation

A variety of methods were used to annotate genes. blastp (v2.2.31+) was used to annotate homologous genes in NR, UniProt Sprot, KOG, and TAIR databases. Interproscan (v5.25–64.0) was also used to predict gene functional domains. Conserved domains were searched using rpsblast (v2.2.31+) with the NCBI CDD. KEGG orthology pathway identification was performed using KEGG software^57^. The protein-protein interaction (PPI) network was obtained based on homology alignment of the STRING database (*Arabidopsis thaliana* v10.5)^58^. For each transcript, the hit with lowest E-value was considered its functional annotation. The annotation of the longest transcript for a gene was used as the annotation for the gene.

### Comparative analysis of gene annotations

IGIA annotation was compared with existing gene annotations and current methods. NGS-based transcripts were predicted using Cufflinks (v2.2.1), Stringtie, and TACO (v0.7.2) with default parameters. Pacbio-based assembly was predicted using Pacbio ToFU pipeline (v2.2.1) with default parameters. Because Cottongen gene annotation was not available for new versions of the cotton genome *G. arboreum*, the Cottongen sequences^15^ were aligned to the new genome as gene annotations using STARlong with the same parameters as on the Pacbio Iso-seq data. Cottongen and CGP assemblies were selected as current annotations. For junction comparison, junctions in different gene annotations that overlapped but did not appear in the other’s annotation were defined as conflicting junctions and were used for subsequent experimental validation.

### Expression and tissue specificity analysis

The IGIA core gene expression were quantified using Stringtie. To describe the tissue specificity of a gene in different tissues, an entropy-based method was used to calculate tissue-specific score *S* as follows:

1 $$S = H_{\mathrm{max}} - H_{\mathrm{obs}} = \mathrm{log}_{2}N - \left( { - \mathop {\sum }\limits_{i = 1}^{N} P_{i} \times \mathrm{log}_{2}P_{i}} \right)$$

where *P*_*i* _is the relative level in tissue *i*. For a gene, if the expression level of tissue *i* was higher than 20% of the highest expression, the gene was considered expressed in tissue *i*.

### Alternative TSS and TES analysis

In order to obtain more comprehensive variations of UTR regions caused by alternative TSS/TES usage, the TSS and TES cluster with average TPM > 0.5 in 16 tissues were used in this part of analysis. The TSS clusters with TPM > 0.5 in at least two tissues were used in a published protocol^59^. Here, the same threshold was used, but to obtain more credible results, an average expression value in all samples was required to pass the filter. For any two clusters within 50 nt with each other, only the one with larger signal was used for subsequent analysis.

For a gene with multiple TSSs, the dominated TSS in one tissue was defined as the TSS having more than 50% CAGE-seq signal across the gene. In analyzing differential TSS usage across multiple tissues, a dynamic TSS switch gene was identified by the two criteria: it has two or more dominant TSSs in any two tissues and the TSS switch score is >0.3. The TSS switch score *W* for tissue *i* and tissue *j* was defined as:

2 $$W_{i,j} = \left\{ {\begin{array}{*{20}{l}} {P_{i,d\left( i \right)} + P_{j,d\left( j \right)} - 1,} \hfill & {{\mathrm{if}}\;d\left( i \right) \ne d\left( j \right)} \hfill \\ {0,} \hfill & {{\mathrm{else}}} \hfill \end{array}} \right.$$

where *d*(*i*) is the dominant TSS site in tissue *i*, *P*_*i,s*_ is the CAGE-seq signal usage of site *s* in tissue *i*.

To investigate the pattern of 3′UTR usage, the weighted 3′UTR length metric was calculated as in published study^60^. In addition, the genes with an alternative 3′UTR between two samples were defined by a change of weighted 3′UTR length >100 nt and TES expression with TPM > 5 in both samples.

For alternative TSS and TES function analysis, the sequences of alternative 5′UTRs and 3′UTRs were extracted to perform the following analyses: miRNA target site prediction using psRNATarget^61^, RG4 motif scan with qgrs in which a motif with more than three G4 planes and a link sequence length greater than one ribonucleotide was accepted, U-rich motif search using Biopython, uORF search using ORFfinder. The 3D structures of long and short isoforms of NRT1.2 were built using SWISS-MODEL^62^ homology modeling and visualized with pymol (v2.2 at https://www.pymol.org).

### AS analysis

The AS events were identified using rMATS (v3.2.5). For each transcript, the CDS sequence was extracted and translated to predict its functional domains. The conserved domain and Pfam domain were searched using rpsblast (v2.2.31+) in the CDD of NCBI and PfamScan (v1.6) of EBI, respectively. The Slim region, disorder region, signal peptide, and TM helices were predicted using iupred (v1.0), VSL2, and anchor (v1.0), signalP (v4.1), and TMHMM (v2.0c), respectively. Subcellular localization with a score > 0.9 in TargetP (v1.1) was chosen. GO term enrichment was analyzed using DAVID web server (v6.8)^63^.

### AS hotspot model

A statistical model was designed to predict the AS hotspot phenomenon. In this model, one gene was divided into several segments based on the exon boundaries. For each isoform of one gene, a string of ones and zeros can be produced to represent its structure, if “1” is defined as the appearance of a segment, “0” as the absence of a segment (Supplementary Fig. 10e).

Based on the string matrix of multiple isoforms for all genes, whether there was AS hotspot in a genome could be estimated by counting the types of substrings (Supplementary Fig. 10f) of annotated isoforms for statistical test. If there was no AS hotspot, the null hypothesis was that AS events were independent of each other and the probability for the number of isoform substrings with given length followed the Markov property.

Under this hypothesis, the number of k-length isoform substring was subject to the distribution *X*:

3 $$P\left( {X\left( {\xi = n{\mathrm{|}}K = 1} \right)} \right) = \left\{ {\begin{array}{*{20}{l}} {p,} \hfill & {{\mathrm{if}}\;n = 2} \hfill \\ {1 - p,} \hfill & {{\mathrm{if}}\;n = 1,} \hfill \\ {0,} \hfill & {{\mathrm{else}}} \hfill \end{array}} \right.$$

4 $$P\left( {X\left( {\xi = n{\mathrm{|}}K = k + 1} \right)} \right) = \mathop {\sum }\limits_{i = \frac{n}{2}}^n C_i^{n - i}p^{n - i}(1 - p)^{2i - n}P\left( {X\left( {\xi = i{\mathrm{|}}K = k} \right)} \right)$$

where $\xi$ was path number variable, *K* was the substring length variable. The expectation of *X* and the probability *p* satisfied $E(X) = (1 + p)^K$, which could be used to estimate *p* and the distribution *X*. The presence of AS hotspot in a genome was called when the observed probability was three-fold larger than the expected distribution under the null hypothesis. The minimum number of isoform substrings for that difference was defined as the cutoff for identifying AS hotspot region.

The above-described model was used to scan the genomes of *G. arboreum*, *A. thaliana*, *H. sapiens*, and *M. musculus* with k-mer from 3 to 10 (Supplementary Fig. 10g and Fig. 4h). To reduce the noise, only the AS hotspots supported by more than three evidences were used for further analysis.

### Polycistron analysis

Polycistron was defined as a transcript containing more than one major CDS region. GO similarity was computed using GOSemSim (3.4.1). KO pathway identification with KEGG software was used for pathway analysis. The protein–protein interaction network obtained based on homology alignment of the STRING database (*Arabidopsis thaliana* v10.5) was used for protein interaction analysis. To examine the co-linearity of polycistrons in other plant genomes, a CDS pair in cotton polycistron was defined to be co-linear in another species if their orthologs were located on the same strand of the same chromosome and the distance between them was less than 20 Kb. Since the genome *G. raimondii* had close relationship to our *G. arboreum* genome, we used blastp (e-value <1e-6) to map the orthologs, while for other plant species the orthologs were obtained from the PLAZA dicots database (v4.0 at https://bioinformatics.psb.ugent.be/plaza).

### PCR validation

Total RNA was treated with DNase I (Fermentas) to digest genomic DNA and then was reverse-transcribed to cDNA using SuperScript III (Life Technologies) with oligo dT or random primers. For validating the JS errors, the pooled cDNA sample was used and primers were designed to span the junctions using Primer 5 software. The PCR products were gel-purified and Sanger-sequenced. For validating the tissue-specific expression of genes, the SYBR Green quantitative real-time PCR (qPCR) analysis was used. The housekeeping genes predicted based on the transcriptome across 16 tissues were validated and selected as internal controls for qPCR. For TSS and TES validation assays, 5′ and 3′RACE strategies were applied for cDNA synthesis and reverse transcription PCR (RT-PCR) amplification, respectively. All primers used in this study are listed in Supplementary Data 4, 5, 11, 14, 31 and 36.

### Nitrate uptake assay

The stably transfected HEK293T cell lines with pMSCV-puro plasmid expressing the long and short versions of coding sequence Ga12g01154 (*NRT1.2*): *NRT-L* and *NRT-S* respectively, and pMSCV-puro empty plasmid as control were cultured in DMEM medium. For measuring NO_3_^-^ uptake activity, the isotope ^15^NO_3_^-^ (10 mM) was added to the medium. The uptake was terminated by removing the uptake solution after 2 and 8 h, respectively, and followed by washing with ice-cold PBS twice. Then, the cells were lyophilized and transferred for LC-MS/MS analysis. The transport activity of NO_3_^-^ was calculated by the ratio of isotope nitrate uptake to cell dry weight.

### EMSA assay

The AP2 domains of AP2-I (235–318 aa) and AP2-S (235–303 aa) were expressed with C-terminal 6xHis tag in *E.coli* BL21 and purified with an Ni-NTA column. The 58 nt DNA probe containing the binding motif (ATGTCGAT) of APETALA (AP2) orthologue in *Arabidopsis*^64^ was used to test the binding activity. The binding reaction (20 μL) consisted of 0.5 μg protein, 2 μL 10x binding buffer (100 mM Tris-HCl, 0.5 M KCl, 10 mM DTT, pH 7.5), 1 μL 50% glycerol, 1 μL 1% NP40, 1 μL 1 M KCl, 4 μL 25 mM MgCl_2_, 0.1 μg Poly(dI-dC), 5 pmol FAM-labeled (hot) probe, ±250 pmol unlabeled (cold) probe. The reaction was incubated at 25 °C for 60 min and electrophoresed on 8% native PAGE gels. Finally, FAM fluorescence signals were detected using the Bio-Rad ChemiDoc MP Imaging system. Probes are listed in Supplementary Data 31.

### **In situ** hybridization

According to the DIG RNA labeling kit protocol (Roche), T7 RNA polymerase mediated in vitro transcription was used to produce digoxigenin-labeled sense and anti-sense probes. The cotton ovules (0, 3, and 5 DPA) were fixed with 4% paraformaldehyde, dehydrated with ethanol series ranging from 15% to 100%, and embedded in paraffin. Next, 14 μm sections were prepared and hybridized with probes. The hybridization signals were produced with anti-digoxigenin-AP and NBT/BCIP kit (Roche) and detected under light microscope. Primers are listed in Supplementary Data 11.

### SNP and GWAS data

SNP data for *G. arboreum* genome were obtained from a previous study^7^. The GWAS sites were obtained from the studies in *G. arboreum*^7^ and *G. hirsutum*^65–70^. For the GWAS data in *G. hirsutum*, only the GWAS sites in At-subgenome were used and mapped to *G. arboreum* genome (AA). For a GWAS site *S*_*T*_ in At, the following procedure was implemented to locate the corresponding *S*_*A*_ in AA genome. The sequence ±1000 bp flanking *S*_*T*_ was mapped to AA genome using STARlong, and the uniquely mapped hit was considered potential region containing *S*_*A*_. The sequence ±200 bp flanking *S*_*T*_ was mapped to the region using BLAT, and the corresponding position to *S*_*T*_ in the alignment was considered *S*_*A*_ if the alignment did not have mismatch ±50 bp flanking *S*_*A*_ and did not have any indel. The number and frequency of SNPs and GWAS sites in intervals were computed using BEDTools (v2.26.0). SNP profile around certain intervals was computed with 5-bp smoothing window.

### Statistical analysis

The majority of statistical tests were conducted using Wilcoxon rank sum test with continuity correction (two-tailed). The pathway analysis and protein–protein-interaction in polycistron analysis was one-tailed Wilcoxon rank sum test, and analysis of the activity difference between NRT-L and NRT-S was a two-tailed *t*-test. The significance levels are indicated by asterisks (*P < 0.05, **P < 0.01, ***P < 0.001). The numbers of TSSs in CAGE signal usage analysis were *n* = 5096/8659/6723 for CT/PT/DT, respectively. In addition, the numbers of TESs in polyA signal usage analysis were *n* = 870/9180/9493 for CT/PT/DT, respectively. For the statistics of 3′UTR length, 5460 genes after removal of polycistrons were used. In length comparison analysis for AS hotspot genes, 135 IGIA cotton genes and 142 *A. thaliana* genes were used. For all the polycistron analyses, the sizes of bi-cistrons and tri-cistrons were 1081 and 34, respectively. The random control sets had matched sizes. In all boxplot plots, the 5%, 25%, 75 and 95% quantiles are shown, and the center line represents the median value.

### Reporting summary

Further information on research design is available in the Nature Research Reporting Summary linked to this article.

## Supplementary information

Supplementary Information

Peer Review

Reporting Summary

Description of Additional Supplementary Files

Supplementary Dataset 1-39

Source Data

## Data Availability

Data supporting the findings of this work are available within the paper and its Supplementary Information files. A reporting summary for this Article is available as a Supplementary Information file. The datasets generated and analyzed during the current study are available from the corresponding author upon request. The sequencing data from this study have been submitted to the NCBI Sequence Read Archive under the accession PRJNA507565. The IGIA gene annotation and all RNA signal tracks generated in this study are freely available in the customized genome browser at http://cotton.whu.edu.cn/igia. The source data underlying Figs. 1e, 2b–d, 2f, 2h, 3b–h, 4e–h, 5b, and 5f–i, as well as Supplementary Figs. 1b, 1d, 2b–f, 4, 5a, 5e, 6d, 6e, 7, 8, 9, 10b, 10g, 11, and 12 are provided as a Source Data file.

## Code availability

The IGIA package is available from GitHub (https://github.com/zhouyulab/igia/). All the softwares used in this study are listed in Supplementary Data^39^.
