# Supplementary material for: Multi-strategic RNA-seq analysis reveals a high-resolution transcriptional landscape in cotton
Source: Nat Commun. 2019 Oct 17;10:4714. doi: 10.1038/s41467-019-12575-x (PMC6797763; doi:10.1038/s41467-019-12575-x)
Supplement: Supplementary file 1 — Supplementary Information [file 41467_2019_12575_MOESM1_ESM.pdf]

Multi-strategic RNA-seq analysis reveals a high-resolution transcriptional landscape in cotton

Wang *et al.*

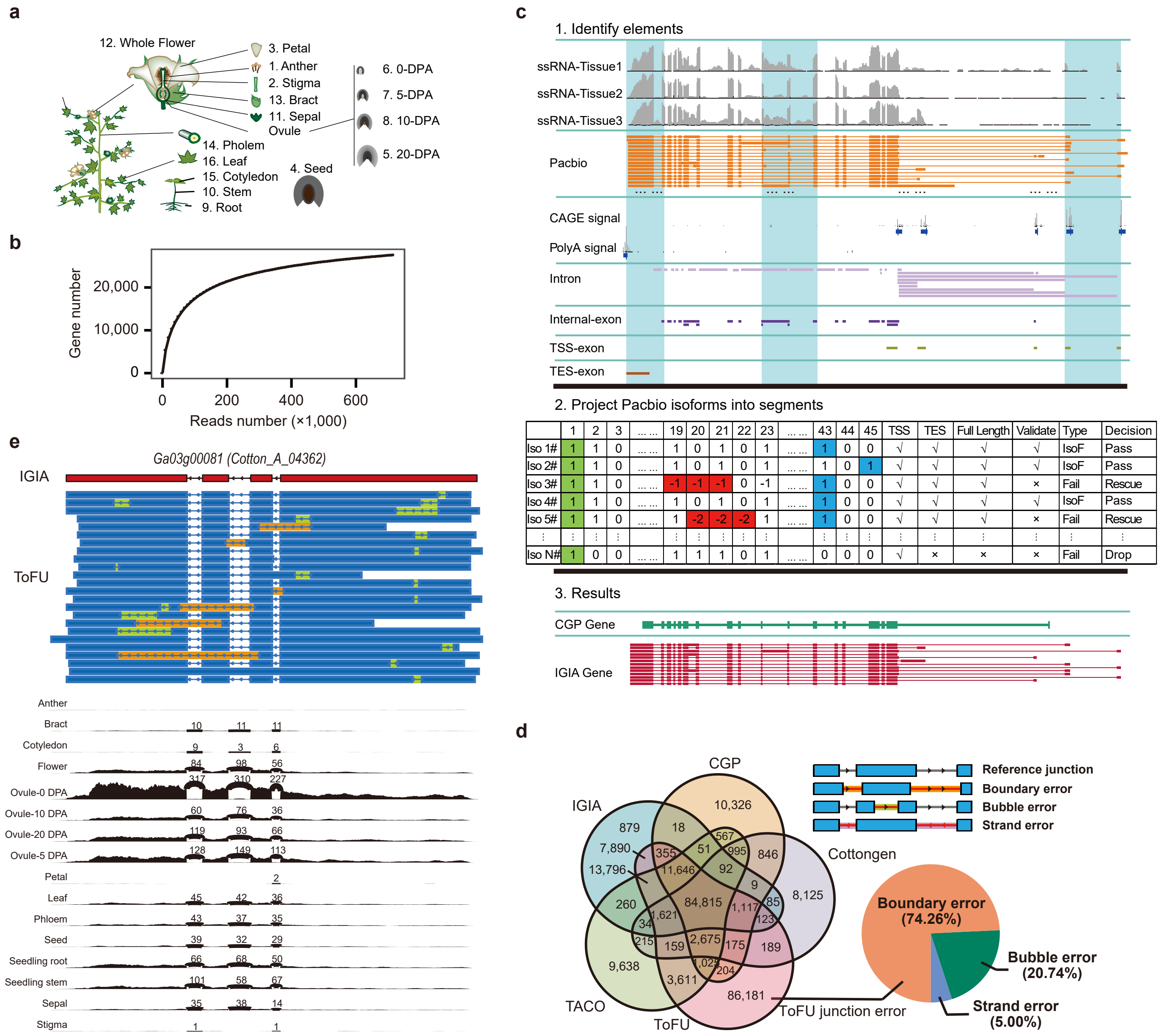

Supplementary Figure 1. IGIA method and error correction from multi-tissue and multi-type data integration. (**a**) Graphical illustration of the 16 tissue samples collected from cotton (*G. arboreum*). (**b**) Gene number saturation analysis for Pacbio reads. The X-axis shows the number of mapped Pacbio long reads; Y-axis shows the number of genes discovered. (**c**) Graphical illustration of IGIA algorithm on one gene example. (**d**) Comparisons of splicing junctions between IGIA, CGP, and Cottongen, and prediction using TACO and ToFU. The graphical illustration (upper right) and statistics (bottom right) of the type of junction errors from ToFU are shown. (**e**) The gene example showing splicing junction errors in ToFU annotation. The regions for junction errors (orange box) and bubble errors (green box) do not have any short read support from NGS ssRNA-seq across 16 tissues. The number above the junction sites represents the number of NGS reads that span splice junctions. The source data underlying Supplementary Figure 1b and 1d are provided as a Source Data file.

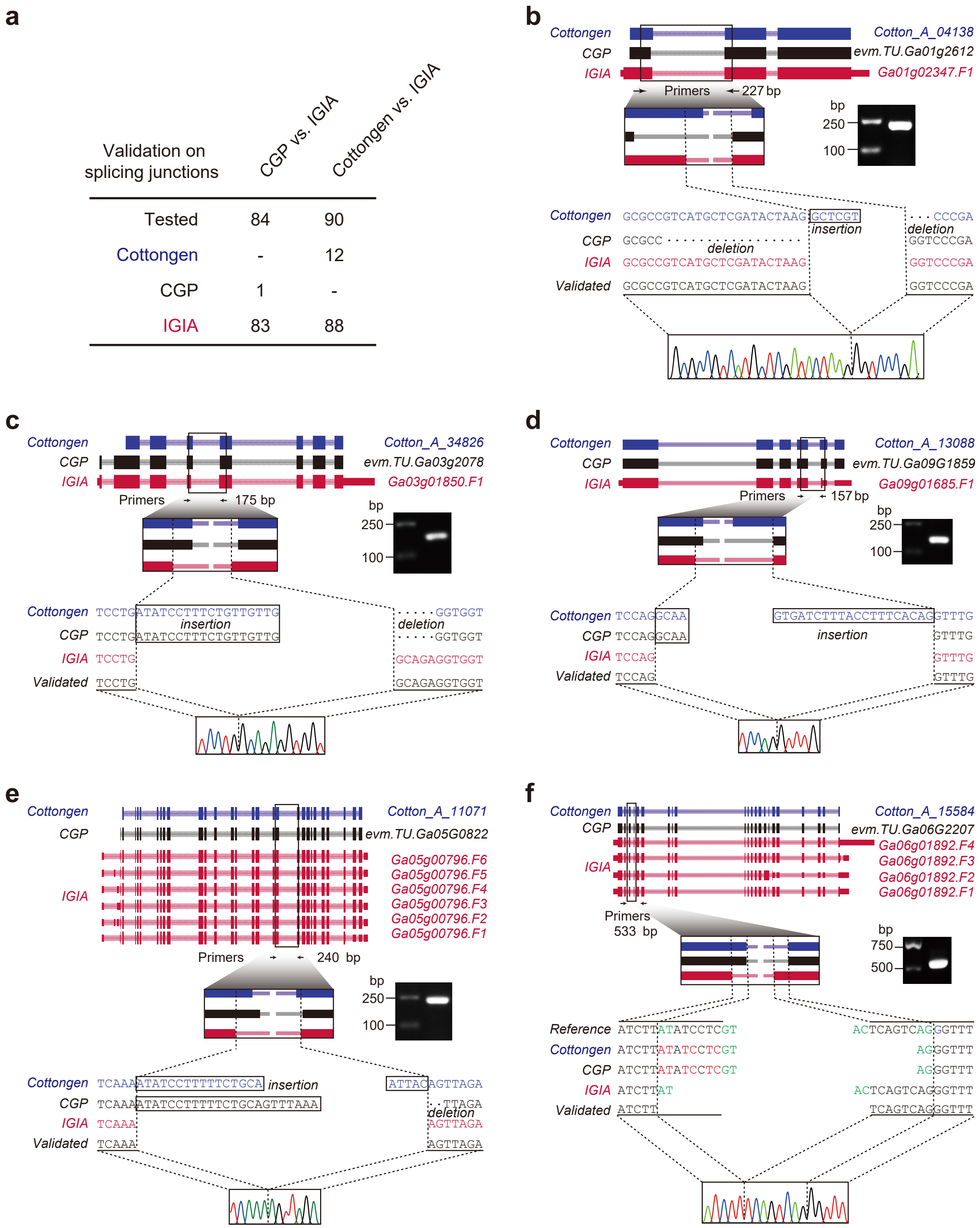

Supplementary Figure 2. RT-PCR validation showing IGIA correctly identified the splicing junction. (**a**) The experimental validation statistics for splicing junction. (**b-f**) Several verification results for the splicing junctions. The Sanger sequencing visualized using Chromas were compared with IGIA, CGP, and Cottongen annotations. The source data underlying Supplementary Figure 2b-f are provided as a Source Data file.

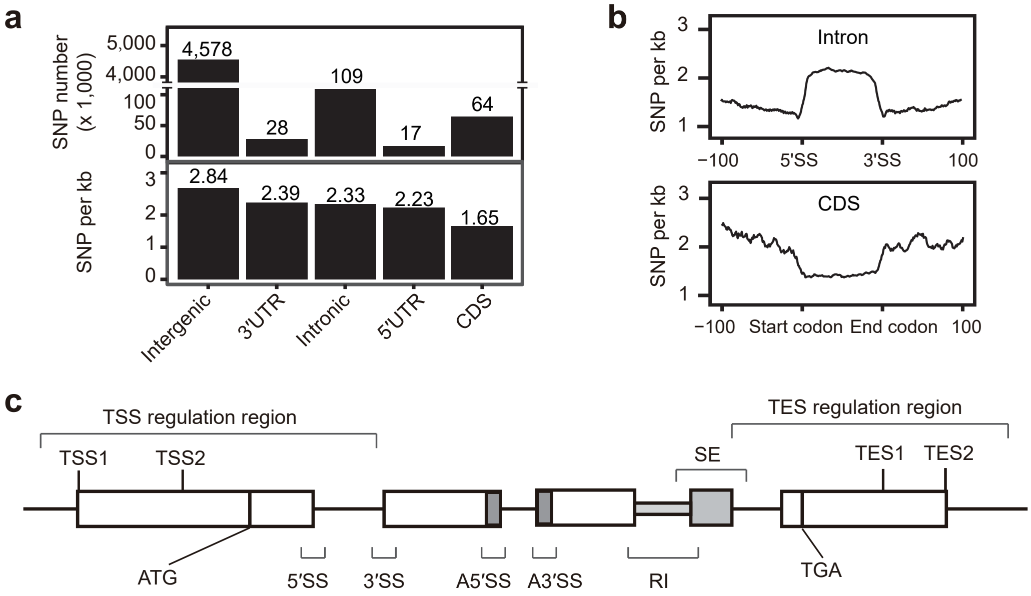

Supplementary Figure 3. The SNP distribution in *G. arboreum* genome. (**a**) The total and region length normalized SNP numbers at different genomic regions including Intergenic, 3′UTR, Intronic, 5′UTR, and CDS. (**b**) The meta-profiles of SNP frequency in intron (top) and CDS (bottom) regions. Their upstream and downstream 100 nt regions are also shown. (**c**) Graphical illustration of different regulatory regions.

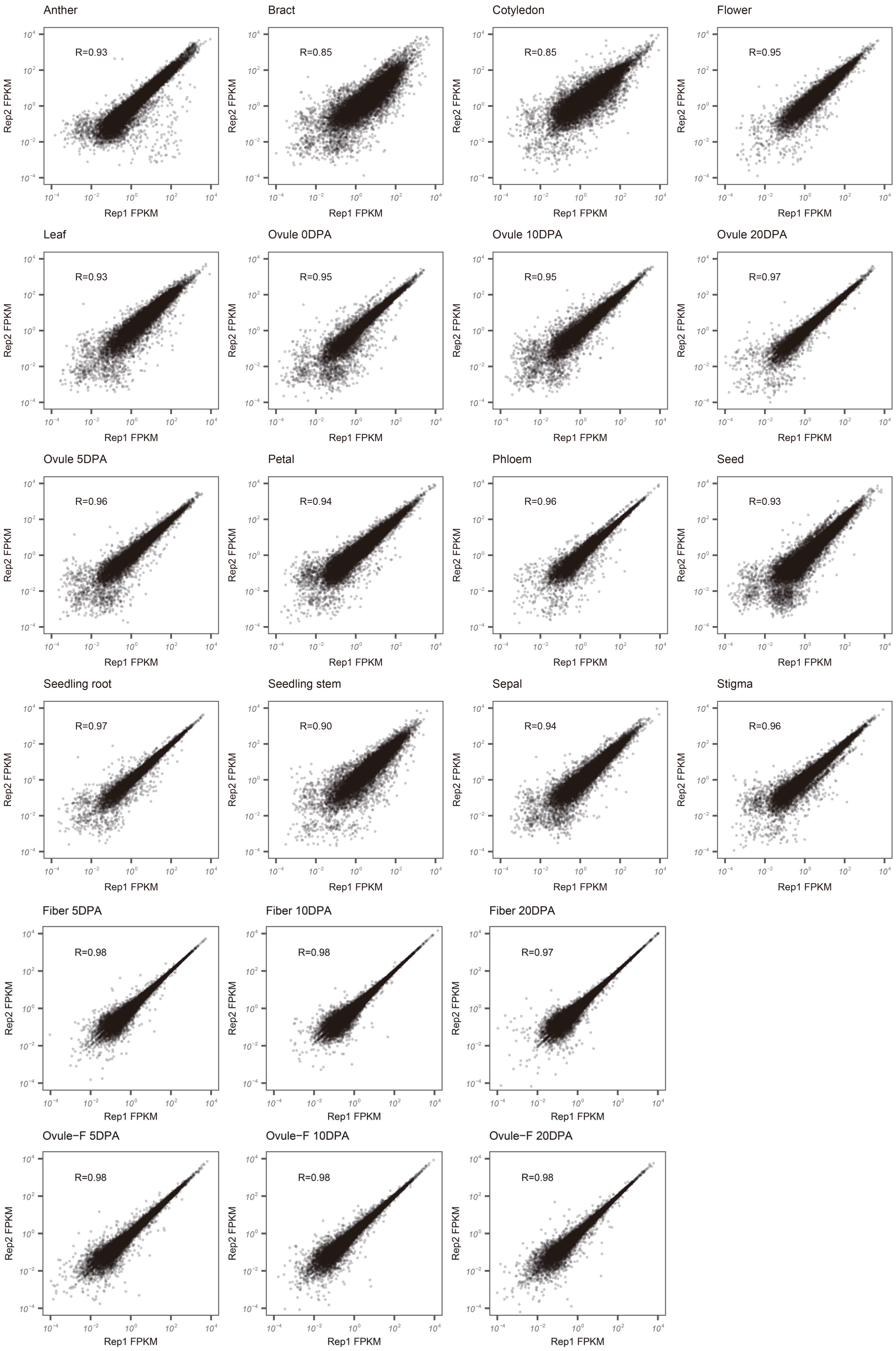

Supplementary Figure 4. Correlations of gene expression between replicated experiments for 22 tissues. The Pearson's correlation coefficients of the gene expression for two biological replicates are shown. Source data are provided as a Source Data file.

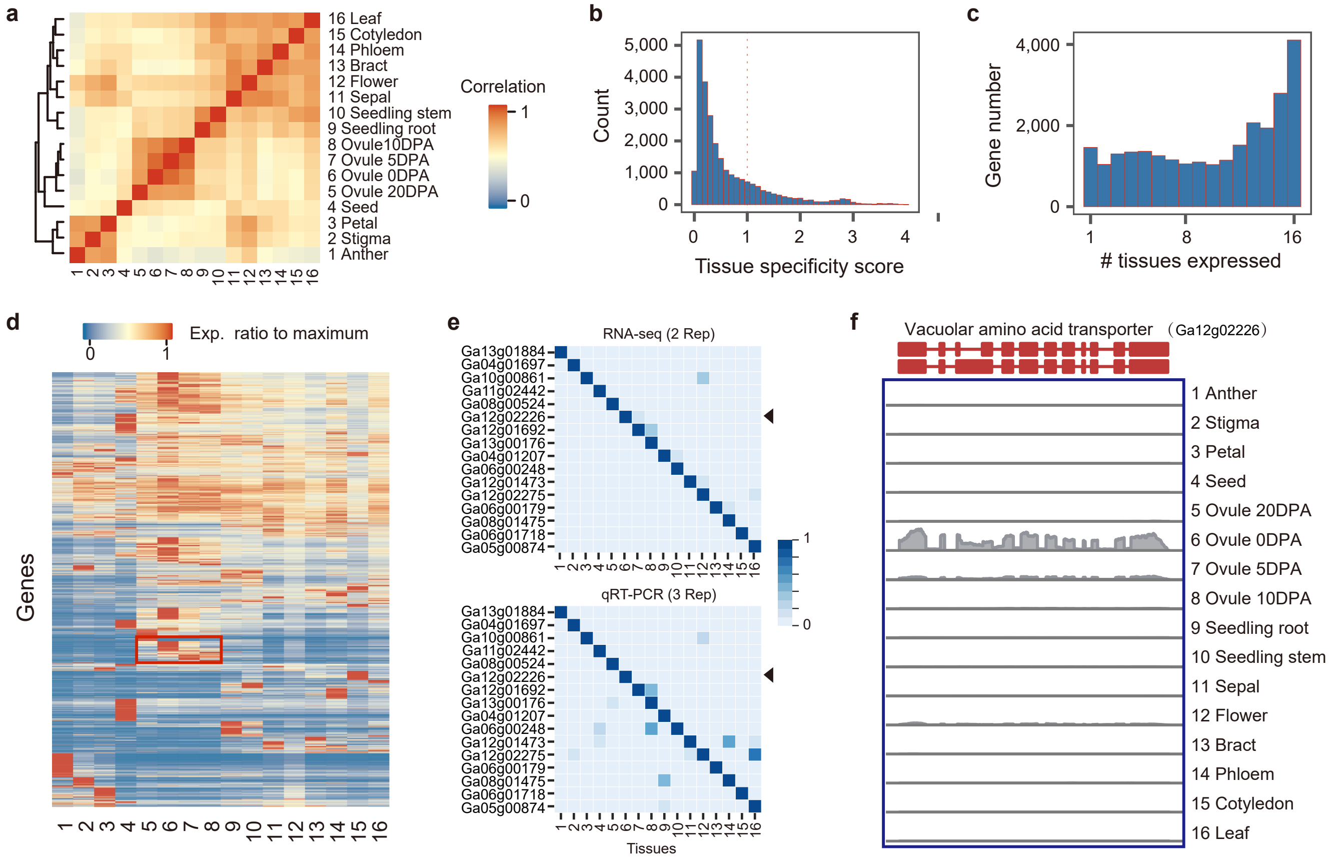

Supplementary Figure 5. Gene expression dynamics across 16 tissues in *G. arboreum*. (**a**) Hierarchical clustering of gene expression correlation across 16 tissues. The color scale 0-1 represents Spearman's correlation coefficients. The numbers 1-16 are abbreviations for the 16 tissues. (**b**) Distribution of the tissue specificity scores of genes. The dashed line indicates the threshold for defining tissue-specific genes with score ≥ 1. (**c**) The number of genes expressed in specific number (from 1-16) of tissues. (**d**) Relative gene expression profile. The gene per row is normalized to its maximum value in 16 tissues. (**e**) Comparison of RNA-seq quantification (top) and qPCR (bottom) of 16 representative tissue-specific genes in 16 tissues. For each gene, the expression values in 16 tissues were normalized to its maximum value. (**f**) ssRNA-seq signal tracks for a tissue-specific gene. The source data underlying Supplementary Figure 5a and 5e are provided as a Source Data file.

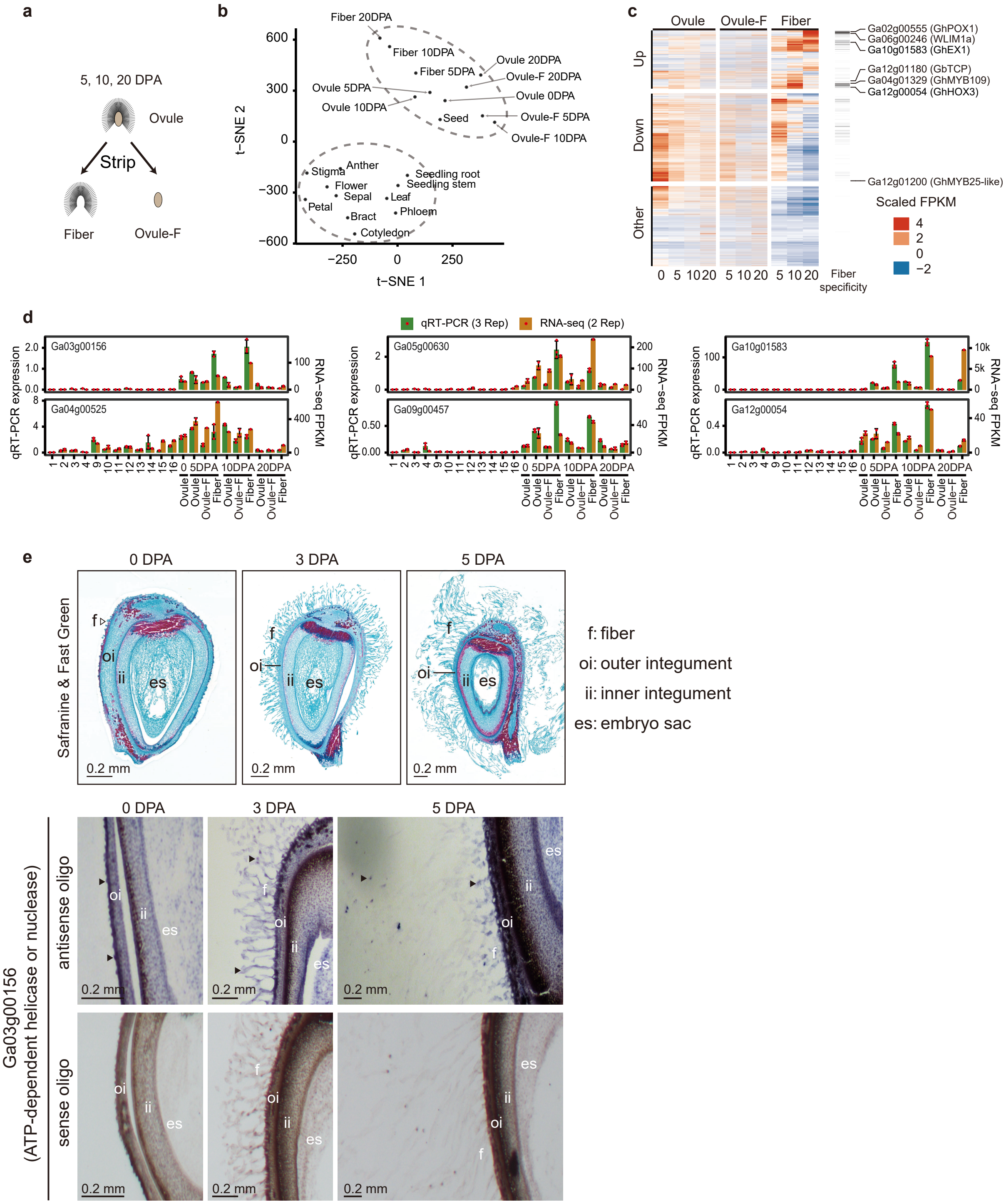

Supplementary Figure 6. Fiber-specific genes in *G. arboreum*. (**a**) Graphical illustration of the separation of ovule and fiber. The ovule without fiber was termed ovule-F, and the stripped fiber was termed Fiber. (**b**) t-SNE plot for 16 tissues and the fiber and ovule-F at 5, 10, and 20 DPA. (**c**) The gene expression heatmap of ovule, ovule-F, and fiber. The fiber-specific genes were marked. (**d**) The RNA expression of 6 fiber-specific genes detected using qPCR and RNA-seq (error bar represents s.d.). (**e**) Expression analysis of Ga03g00156 (ATP-dependent helicase/nuclease) in G. arboreum ovules at 0, 3, and 5 DPA based on in situ hybridization. Top: sections of ovlues at 0, 3, and 5 DPA, stained with safranine and fast green. Bottom: Digoxigenin-labeled antisense and sense oligos were used as probe and control, respectively. The arrowheads marked several fiber cells. Bar = 200 μm. The source data underlying Supplementary Figure 6d and 6e are provided as a Source Data file.

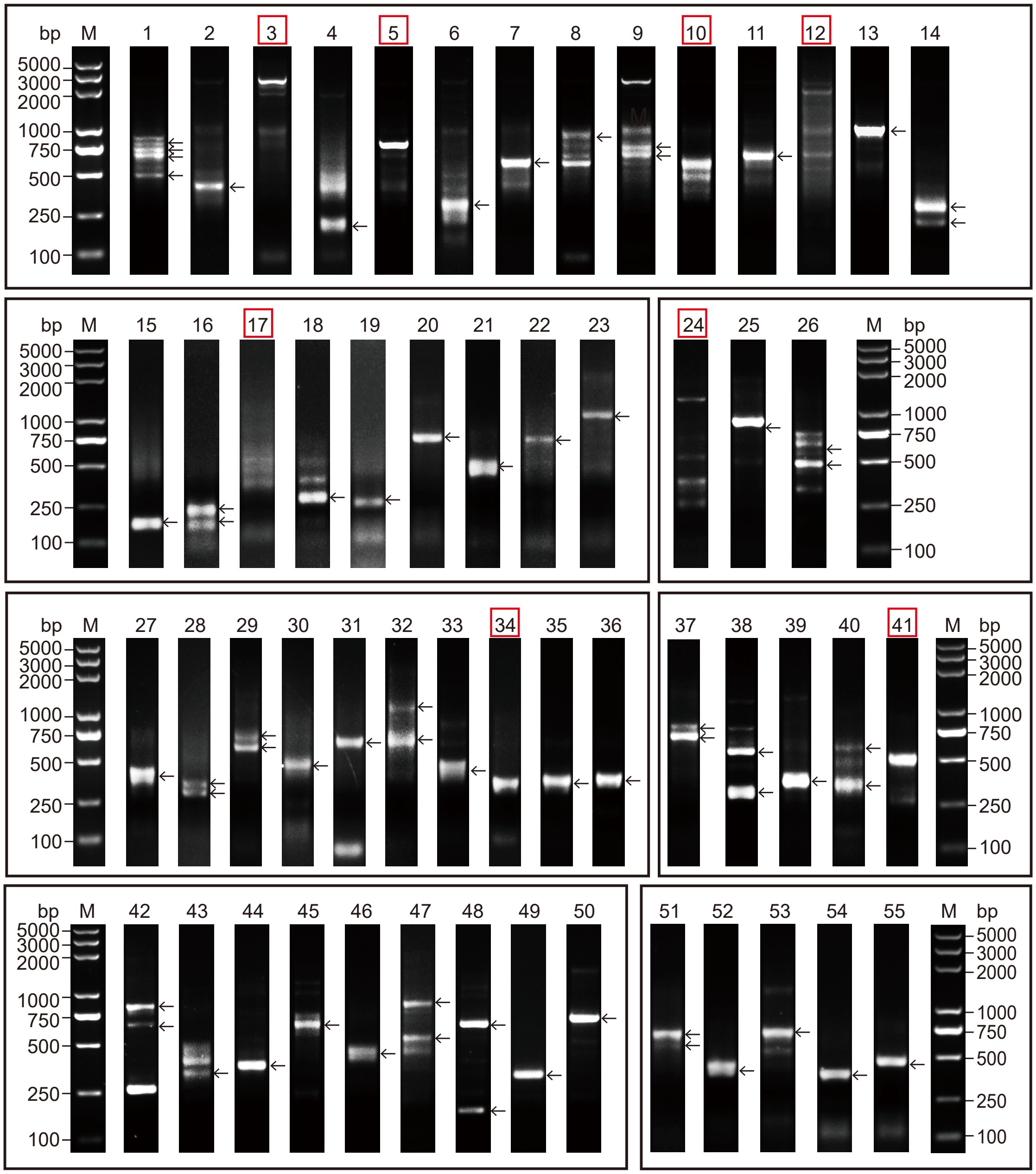

Supplementary Figure 7. 5′RACE-PCR validation of randomly selected TSSs. The expected bands are indicated by arrows and the lanes with putative PCR failure and unspecific bands are marked with red rectangle boxes. The gene and primer information is provided in Supplementary Data 14. Source data are provided as a Source Data file.

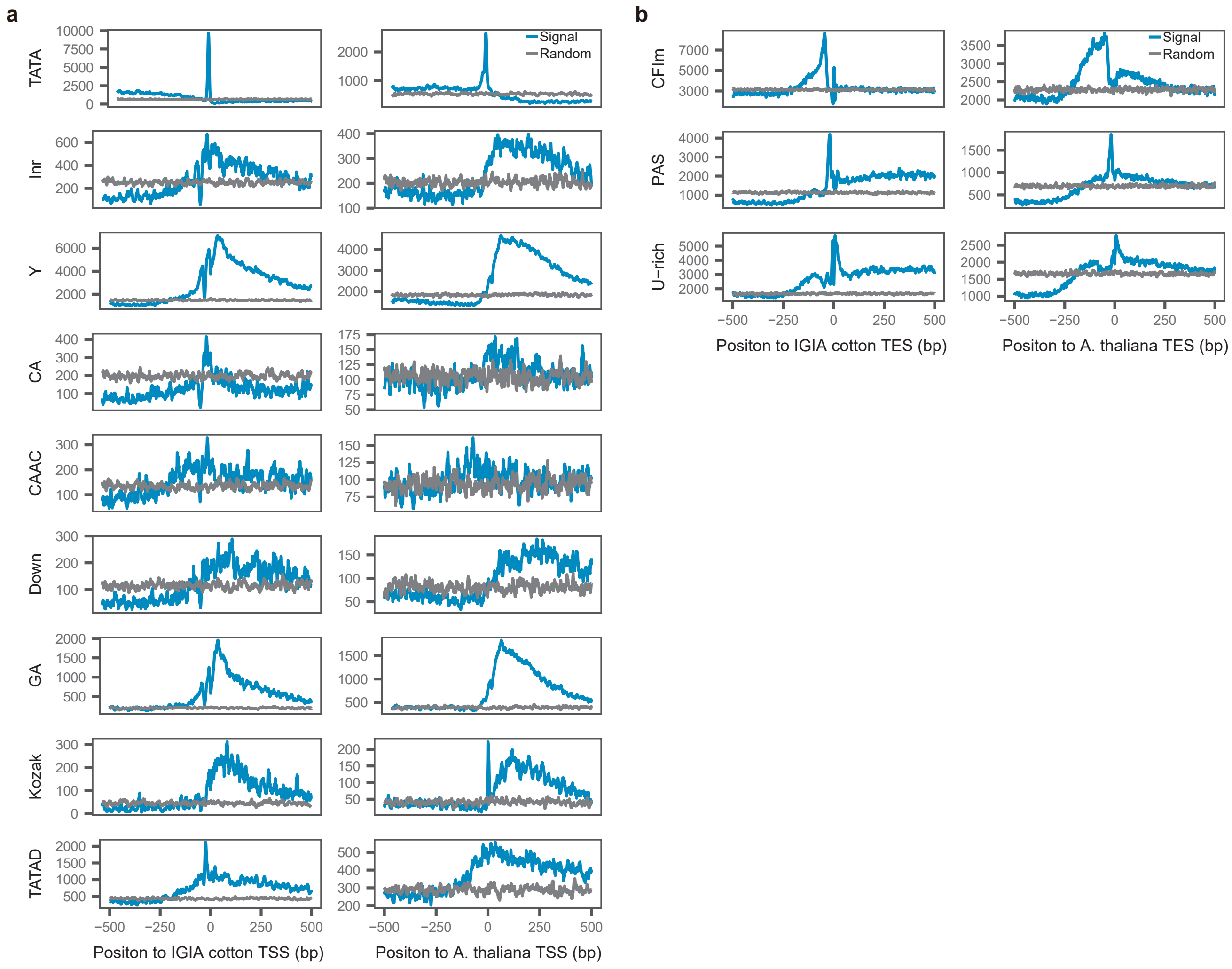

Supplementary Figure 8. The sequence features around TSSs and TESs. (**a**) The known TSS features include CA, CAAC, Down, GA, lnr, Kozak, TATA, TATAD, and Y motifs. (**b**) The known TES features include CFlm, PAS and U-rich motifs. The signals in random controls are shown with gray lines. Source data are provided as a Source Data file.

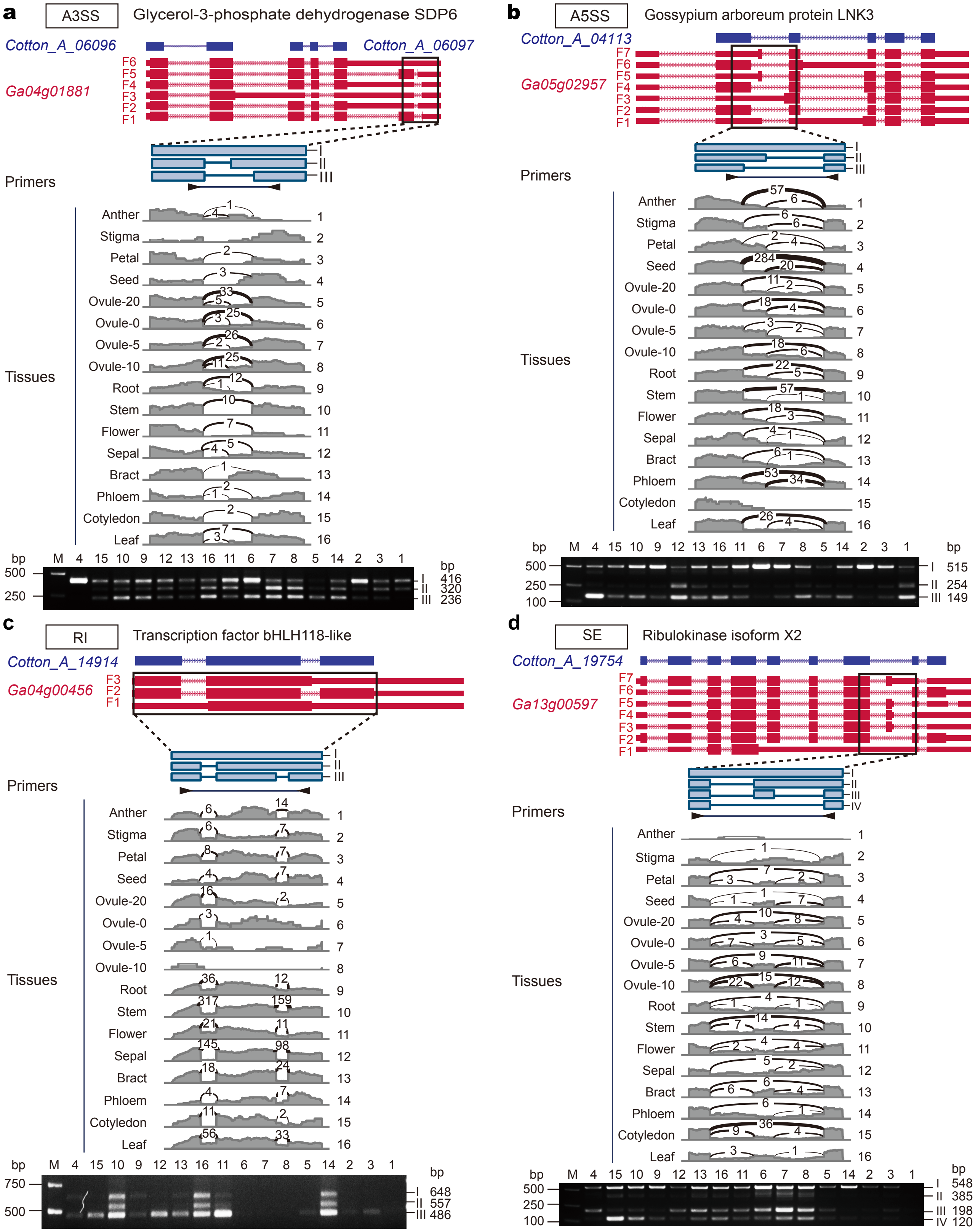

Supplementary Figure 9. RT-PCR validated AS events. The Cottongen annotation, IGIA isoforms, and the ssRNA-seq signals across 16 tissues are shown. The examples of A3SS, A5SS, RI, and SE are shown in (**a**) - (**d**), respectively. Source data are provided as a Source Data file.

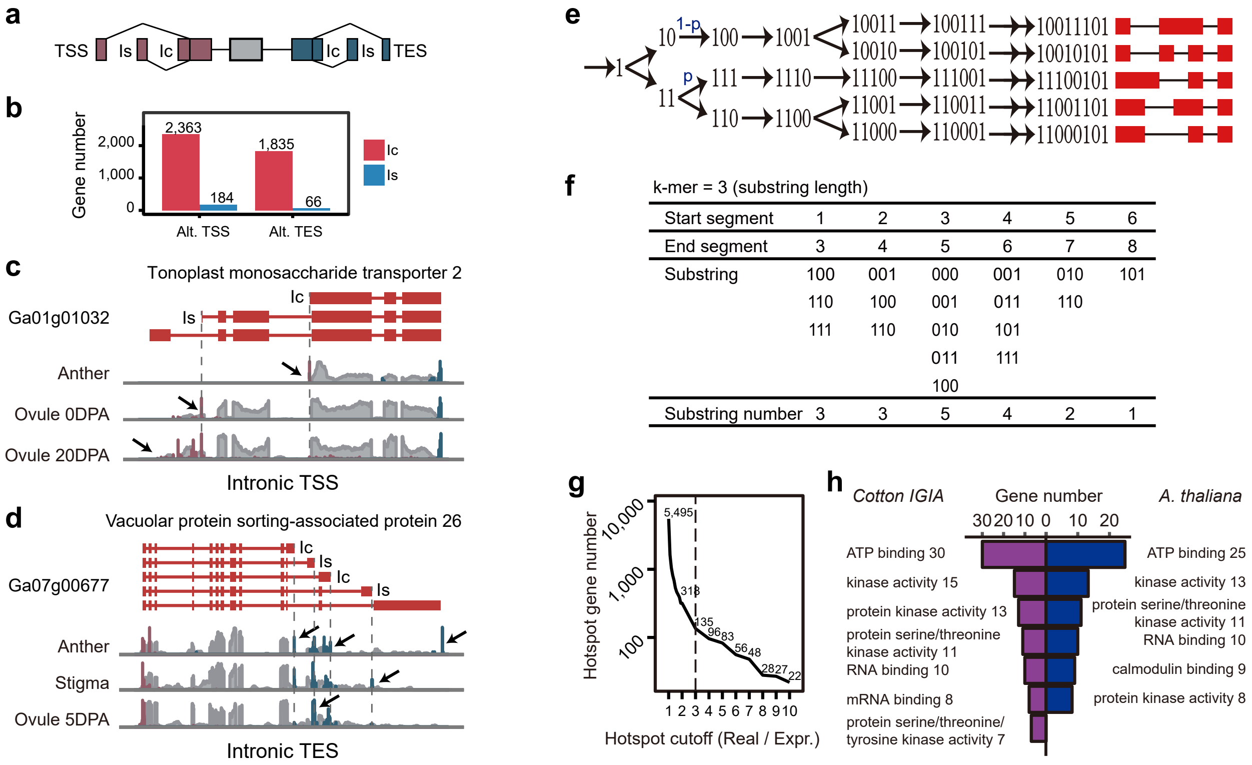

Supplementary Figure 10. Identification of the genes with intronic TSS/TES switching and AS hotspots. (**a**) Schematic illustration of intronic TSS/TES switching events. Is: intronic TSS/TES in skipped terminal exon; Ic: intronic TSS/TES in composite terminal exon. (**b**) Overview of intronic TSS and TES changes in cotton genome. (**c**) A gene example of intronic TSS change. (**d**) A gene example of intronic TES change. (**e**) Computational model for identifying AS hotspots. (**f**) A statistical example with k-mer = 3 based on the model. (**g**) The results of identified hotspots at different cutoffs. Dashed line marks the cutoff used in this study. (**h**) GO term annotations for genes with AS hotspots in *G. arboreum* and *A. thaliana*. The source data underlying Supplementary Figure 10b and 10g are provided as a Source Data file.

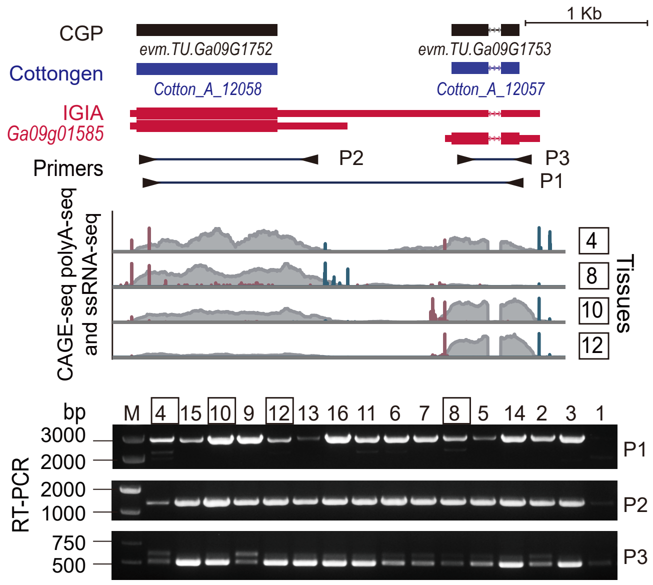

Supplementary Figure 11. Polycistron example with two CDSs. The CGP, Cottongen, and IGIA annotations, CAGE-seq and PolyA-seq signals from mixed tissues, and ssRNA-seq signals in four tissues are shown. RT-PCR validations were performed in 16 tissues. The three probes are indicated below the IGIA isoforms. Source data are provided as a Source Data file.

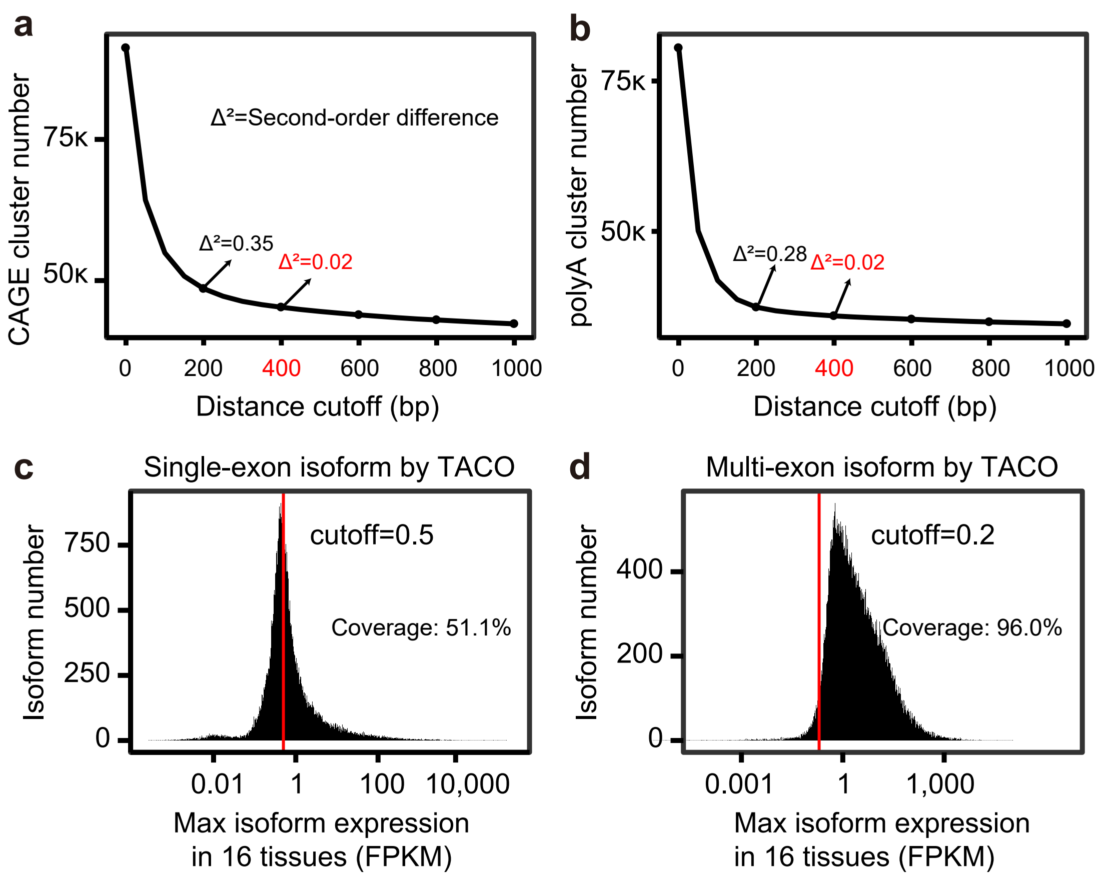

Supplementary Figure 12. The predicted number of CAGE/PolyA cluster and single/multi-exon isoform analysis with different cutoff values. The cutoff values used in this study are highlighted in red. (**a**) The predicted number of CAGE clusters using different distance cutoff values between any two CAGE clusters for one gene. (**b**) The predicted number of polyA clusters using different distance cutoff values between any two polyA clusters for one gene. (**c**) The predicted isoform number using different FPKM cutoff values for single-exon isoforms. (**d**) The predicted isoform number using different FPKM cutoff values for multi-exon isoforms. Source data are provided as a Source Data file.

Supplementary Table 1. Statistics of Pacbio sequencing data

	Cells	Bases (Gb)	Reads	Mean read length	ROIs (Reads Of Insert)	ROI length	ROI quality	Mean No. of passes
<1 Kb	14	25.95	1,362,180	19,051	1,015,633	1048	0.9708	14.73
1-2 Kb	16	22.42	1,236,185	17,458	830,078	1854	0.9737	10.86
2-3 Kb	20	29.03	1,621,526	17,420	995,478	2847	0.9724	9.46
>3 Kb	14	15.51	984,806	15,183	491,525	4174	0.9723	6.83
Total	64	92.91	5,204,697	-	3,332,714	-	-	-

Supplementary Table 2. Statistics of CAGE-seq sequencing data

Tissue	Raw reads	Clean reads	rRNA reads	Mapped	Uniquely mapped	Cluster number
Anther	67,986,394	36,954,976	437,157	32,892,643	26,799,718	21,153
Bract	86,379,263	86,379,263	876,467	75,226,081	39,810,471	31,192
Cotyledon	77,842,989	77,842,989	893,128	66,424,595	28,650,391	24,832
Flower	69,041,744	69,028,937	346,573	56,667,119	30,473,749	30,513
Ovule-0DPA	95,613,710	95,613,710	298,966	89,142,443	43,095,434	33,003
Ovule-10DPA	54,671,800	42,171,951	269,070	38,204,041	26,986,197	26,931
Ovule-20DPA	107,794,533	107,794,533	637,756	95,921,011	40,222,640	26,701
Ovule-5DPA	119,107,602	119,107,602	272,053	110,252,058	48,597,679	32,552
Petal	108,591,308	108,591,308	680,126	96,700,752	44,632,322	29,108
Leaf	98,225,366	98,225,366	1,249,080	86,886,387	40,588,295	30,949
Phloem	108,496,076	108,496,076	701,755	94,943,824	46,372,950	28,872
Seed	86,930,690	86,930,690	1,198,475	72,225,713	30,532,876	16,619
Seedling root	107,978,328	107,978,328	914,823	96,198,027	47,691,930	34,348
Seedling stem	61,043,305	47,413,819	200,705	44,187,726	31,069,209	29,788
Sepal	100,728,978	100,728,978	486,990	91,468,605	37,947,509	27,827
Stigma	70,634,058	36,457,600	154,775	33,683,632	25,898,145	24,453

Supplementary Table 3. Statistics of PolyA-seq sequencing data

Tissue	Raw reads	Clean reads	rRNA reads	Mapped	Uniquely mapped	Cluster number
Anther	123,005,751	87,775,785	363,387	71,999,887	25,591,654	15,224
Bract	184,704,557	57,786,252	24,033	42,729,756	9,615,654	21,241
Cotyledon	282,743,578	83,695,711	95,906	66,553,035	14,114,722	22,061
Flower	171,425,480	90,316,208	16,576	71,190,380	19,840,848	27,508
Ovule-0DPA	141,641,299	35,238,154	15,526	27,955,645	10,778,543	24,204
Ovule-10DPA	145,352,360	76,277,166	24,275	60,815,956	12,703,181	22,651
Ovule-20DPA	229,187,840	60,585,724	43,442	45,573,537	13,770,498	18,620
Ovule-5DPA	130,368,960	45,152,387	16,717	38,052,614	14,598,934	22,402
Petal	135,287,380	72,909,716	17,300	61,835,391	25,437,477	21,732
Leaf	150,513,065	52,878,406	7316	37,785,265	10,951,223	24,590
Phloem	155,611,731	54,198,293	32,057	42,549,704	17,559,072	24,625
Seed	239,639,343	87,897,152	193,379	64,118,269	13,854,251	16,566
Seedling root	150,595,760	56,655,067	70,326	40,916,740	9,584,292	24,168
Seedling stem	205,974,889	96,135,263	143,407	74,421,566	13,947,537	26,432
Sepal	139,910,466	68,878,692	11,017	51,947,077	10,573,125	24,188
Stigma	132,752,994	74,304,029	45,136	62,320,698	21,287,094	19,186

Supplementary Table 4. Genomes used in this study

Species	URL	Version
A. thaliana	https://www.arabidopsis.org	Araport11
O. sativa	http://rice.plantbiology.msu.edu	MSU 7.0
D. melanogaster	https://www.ncbi.nlm.nih.gov/genome/	GCF_000001215.4_Release_6_plus_ISO1_MT
H. sapiens	https://www.ncbi.nlm.nih.gov/genome/	GCF_000001405.37_GRCh38.p11
M. musculus	https://www.ncbi.nlm.nih.gov/genome/	GCF_000001635.25_GRCm38.p5
G. raimondii	https://phytozome.jgi.doe.gov	Gossypium raimondii v2.1
G. hirsutum	https://phytozome.jgi.doe.gov	Gossypium hirsutum v1.1

Supplementary Table 5. Counts of SNPs and GWAS sites in different regulatory regions

Element	Location	SNPs	SNPs per kb	GWAS	GWAS*
TSS	Upstream 1kb	129,899	4.27	42	41
	First exon	24,049	2.00	47	29
	First intron	27,100	2.51	14	14
	Alt 5′UTR	6,090	1.78	5	4
TES	Downstream 1kb	113,948	3.78	47	42
	Last exon	37,117	2.12	88	54
	Last intron	26,687	2.79	21	21
	Alt 3′UTR	14,210	2.68	15	14
ATG	Upstream 50bp	3,621	2.04	7	7
	Start codon	76	0.71	0	0
	Downstream 50bp	2,771	1.56	9	6
TGA	Upstream 50bp	2,849	1.66	7	4
	End codon	155	1.50	0	0
	Downstream 50bp	3,658	2.14	6	6
CS	3′SS (-50∼+50)	23,642	1.63	51	39
	5′SS (-50∼+50)	23,585	1.63	54	37
AS	A3′SS	952	1.85	1	1
	A5′SS	964	1.76	0	0
	RI	10,358	2.02	17	10
	SE	4,199	2.29	1	1

* GWAS sites after removal of those causing amino acid changes
