# Supplementary material for: Multi-strategic RNA-seq analysis reveals a high-resolution transcriptional landscape in cotton
Source: Nat Commun. 2019 Oct 17;10:4714. doi: 10.1038/s41467-019-12575-x (PMC6797763; doi:10.1038/s41467-019-12575-x)
Supplement: Supplementary file 4 — Description of Additional Supplementary Files [file 41467_2019_12575_MOESM4_ESM.docx]

**Description of Additional Supplementary Files**

File name: Supplementary Data 1.

Description: Statistics of ssRNA-seq sequencing data.

File name: Supplementary Data 2.

Description: Genomic coordinates of IGIA gene set in BED format.

File name: Supplementary Data 3.

Description: Gene annotation for IGIA gene set.

File name: Supplementary Data 4.

Description: PCR primers for validation of junction site errors in CGP annotation.

File name: Supplementary Data 5.

Description: PCR primers for validation of junction site errors in Cottongen annotation.

File name: Supplementary Data 6.

Description: PCR validation for splicing junctions (IGIA vs. CGP).

File name: Supplementary Data 7.

Description: PCR validation for splicing junctions (IGIA vs. Cottongen).

File name: Supplementary Data 8.

Description: Expression quantification in RPKM for IGIA genes across 16 tissues.

File name: Supplementary Data 9.

Description: Tissue-specific genes (S ≥1) and their expression values in RPKM.

File name: Supplementary Data 10.

Description: Ovule and fiber specific genes (S ≥1) and their expression values in RPKM.

File name: Supplementary Data 11.

Description: Primers for tissue-specific genes in ssRNA-seq.

File name: Supplementary Data 12.

Description: The counts of CAGE-seq reads in TSS clusters across 16 tissues.

File name: Supplementary Data 13.

Description: The counts of CAGE-seq reads in gene loci across 16 tissues.

File name: Supplementary Data 14.

Description: Primers for 5′RACE validation of CAGE-seq data.

File name: Supplementary Data 15.

Description: Global alternative TSS pattern across 16 tissues.

File name: Supplementary Data 16.

Description: The effects of alternative TSS usage on 5′UTR and CDS.

File name: Supplementary Data 17.

Description: The subcellular localization for full and N-terminal truncated proteins.

File name: Supplementary Data 18.

Description: The counts of PolyA-seq reads in TES clusters across 16 tissues.

File name: Supplementary Data 19.

Description: The counts of PolyA-seq reads in gene loci across 16 tissues.

File name: Supplementary Data 20.

Description: Global alternative TES usage across 16 tissues.

File name: Supplementary Data 21.

Description: The effects of alternative TES usage on 3′UTR.

File name: Supplementary Data 22.

Description: Weighted 3′UTR lengths in 16 tissues.

File name: Supplementary Data 23.

Description: The genomic coordinates and quantification of A3SS events.

File name: Supplementary Data 24.

Description: The genomic coordinates and quantification of A5SS events.

File name: Supplementary Data 25.

Description: The genomic coordinates and quantification of RI events.

File name: Supplementary Data 26.

Description: The genomic coordinates and quantification of SE events.

File name: Supplementary Data 27.

Description: The effects of A3SS on protein features.

File name: Supplementary Data 28.

Description: The effects of A5SS on protein features.

File name: Supplementary Data 29.

Description: The effects of RI on protein features.

File name: Supplementary Data 30.

Description: The effects of SE on protein features.

File name: Supplementary Data 31.

Description: Primers for validation on alternative splicing (AS).

File name: Supplementary Data 32.

Description: The genomic coordinates of microexons.

File name: Supplementary Data 33.

Description: The list of genes with AS hotspots in *G. arboreum*.

File name: Supplementary Data 34.

Description: Pfam domain of cotton hotspot genes.

File name: Supplementary Data 35.

Description: Pfam domain of *Arabidopsis* hotspot genes.

File name: Supplementary Data 36.

Description: Primers for validation of polycistrons.

File name: Supplementary Data 37.

Description: The genomic coordinates of the polycistrons.

File name: Supplementary Data 38.

Description: The GWAS sites in *G. arboreum* genome integrated from different studies.

File name: Supplementary Data 39.

Description: Softwares used in this study.
