# Supplementary material for: Multi-strategic RNA-seq analysis reveals a high-resolution transcriptional landscape in cotton
Source: Nat Commun. 2019 Oct 17;10:4714. doi: 10.1038/s41467-019-12575-x (PMC6797763; doi:10.1038/s41467-019-12575-x)
Supplement: Supplementary file 2 — Peer Review [file 41467_2019_12575_MOESM2_ESM.pdf]

Reviewers' comments:

Reviewer #1 (Remarks to the Author):

In this manuscript, the authors have essentially sought to integrate a variety of sequencing data modalities including Iso-seq, ssRNA-seq, CAGE-seq and PolyA-seq to extensively characterize the transcriptome landscape of Gossypium arboreum, a diploid cotton genome. In addition to tissue-specific gene expression, they investigated features such as transcription start sites, alternative polyadenylation, alternative splicing events and SNPs including those outside coding regions. They have devised an approach IGIA (integrative gene isoform assembler) that allows a better integration of PacBio Iso-seq and NGS based ssRNA-seq, CAGE-seq and polyA-seq than any other existing tools. More specifically, they have demonstrated how the integrative/hybrid approach performs better than ToFU for long reads or TACO, StringTie, Cufflinks etc for short reads. This work improves and extends the existing resources such as Cottongen and CGP in the context of G. arboreum and is certainly of value to the cotton community.

The manuscript mentions a number of bioinformatics tools that authors have used for a variety of analyses: RAMPAGE, CPAT, proovread, KAAS, QGRS Mapper, rMATS, ANCHOR, GoSemSim, iupred, VSL2, anchor, signalP, TMHMM and many others. It will be helpful to the readers to have a listing of all the referred tools, along with a brief description of their main functionality, related publication and download site.

The authors have used sound statistical analyses throughout the paper. However, the authors use various values of cutoff for FPKM, TPM etc without explaining the underlying rationale for the choice of cutoff. For example, FPKM >5 when comparing the different approaches for predictions of splicing junction sites; FPKM >0.5 and 0.2 for single and multi-exon fragments respectively to filter out low expressed fragments; average TPM >0.5 to identify TSS and TES selection.

Lines 45-46: Thus, the cotton is one of the most economically important crop plants world-wide and has long been one of major focuses of plant research[1, 2].
>> Cotton is unarguably a very important crop on a global scale but it does not belong to the group of most valuable crops economically or say in production metric, with rice, wheat, soybean, tomatoes, sugarcane, maize, potatoes and grapes well ahead of it in financial global value. For more specific global statistics, authors are encouraged to visit website of the Food and Agriculture Organization of the United Nations. Cotton is, however, one of the most economically important fiber crop plants world-wide.

Lines 47-50: In the past few years, four different cotton genomes have been successfully sequenced and assembled, including two allotetraploid (AADD) Gossypium hirsutum[3, 4, 5] and Gossypium barbadense[5] genomes, and two ancestor diploid Gossypium raimondii (DD)[1, 6] and Gossypium arboreum (AA)[7, 8].
>> Levant cotton (Gossypium herbaceum) is another genome that has been undergone transcriptomic profiling in recent years and is in fact a close relative of Gossypium arboreum.

Lines 79-81: A computational method named GRIT reconstructed gene models from RNA-seq short reads data by integrating CAGE-seq and PolyA-seq data for Drosophila genome, and has obtained better results than Cufflinks based only on RNA-seq data[26].
>> The authors should discuss GRIT in further details, since one of the main product of this manuscript, IGIA, is being advocated as superior to GRIT. The authors should also discuss how IGIA compares with GRIT, both in conceptual design as well as accuracy/performance metric.

Lines 112-113: Integrating the IGIA core isoforms (IsoF and IsoR) and IsoC, and those isoforms from our TACO pipeline
>> Drop ‘our’ from ‘our TACO pipeline’, unless one or more of the authors have contributed to the

development of TACO pipeline.

Line 122: The cumulative fraction curves show that the transcripts from ToFU annotations with Pacbio long reads
>> Citation missing for ToFU: PLoS One. 2015 Jul 15;10(7):e0132628. Doi: 10.1371/journal.pone.0132628. eCollection 2015.

Lines 223-224: Next we analyzed the consequences on protein for a set of 2,800 proteins with alternative TSSs in coding regions.
>> It is unclear what the authors mean by "consequences on protein".

Here are a few minor edits:

Lines 53-55: Accumulating evidence supports that the regulation of alternative transcript isoforms plays pivotal functions on eukaryote development ...
>> … regulation of alternative transcript isoforms plays pivotal role in eukaryote development ...

Lines 61-62: The regulation of RNA transcription and processing significantly thus affects multiple aspects …
>> The regulation of RNA transcription and processing thus significantly affects multiple aspects …

Lines 361-362: These bicistronic mRNA is produced by transcriptional read-through for the two adjacent genes.
>> This bicistronic mRNA is ...

Lines 450-451: However, the biogenesis and functions of AS hotspot in plants, and same questions to polycistrons, are both intriguing issues to be investigated in future studies.
>> It is unclear what authors mean by 'and same questions to polycistrons'

Lines 492-493: The 5' random barcode in Read1 and Read2, polyA stretch in Read1 and polyT stretch in Read2 were trimed.
> 'trimed' is misspelled.

Lines 523-525: For reads that can not perfect with all of the above methods, we will try to improve it by enumerate its exon path like other NGS-based methods, and the enumerated isoform is called Partial information isoform (isoP).
>> The intent of this sentence is not clear. Consider rephrasing it for clarity.

Lines 577-579: A gene which has at least two dominated TSS site and the change of which usage rate greater than 30% of these two site in two tissues is called dynamic TSS swtich gene.
>> Once again, the intent of this sentence is not clear. Consider rephrasing it for clarity. Also, 'TSS swtich gene' is misspelled.

Reviewer #2 (Remarks to the Author):

The authors of this manuscript have used four different sequencing methods (PacBio iso-seq, RNA-seq, CAGE-SEQ and polyA-seq) to analyze the landscape of a diploid cotton G. arboretum transcriptome using RNA from sixteen different tissues. The main contribution of this paper is the annotation of transcripts in this species including 5'UTRs, 3'UTRs with alternative TSS/TES and alternative splicing. The major conclusions of this paper are convincing, but several of their conclusions are confirmatory. For example, a recent global analysis of alternative splicing, poly(A) and fusion transcript analysis using PacBio Iso-seq in another cotton species (Wang et .al., New Phytol.

2018) and other global alternative splicing papers have reported similar conclusions. Alternative splicing in other diploid and tetraploid species has also been reported (Li et al., Mol. Plant 2014; Zhu et al., BMC Genomics 2018). However, the transcription sites (TSSs) in cotton transcripts have not been reported and this paper adds new information in this area. The information presented in this paper, especially the updated gene/transcript annotation would be useful to the researchers in the cotton community particularly those that are working with the diploid species G. arboretum.
I think an interesting topic that could be addressed with the data that the authors have is the relationship between alternative transcription start sites and alternative splicing as well as alternative splicing and transcription end sites. Analysis of their data to address if there is any coupling between these processes would add novelty to this paper and appeal to the broader research community. Also, some comparative analysis of finding on AS and poly(A) results in this work with recently reported results in another cotton paper (Wang et al., New Phytology 2018) should be presented in the discussion.
It was stated that if the TSS clusters are closer to each other (with in 400bp), only the TSS position with the largest signal was chosen as TSS candidates for integration. They applied the same criteria for TES clusters also. What is the basis for this cutoff? Does this underestimate alternate TSSs and TESs?
The authors conclude that G. arboretum genome has unique features including longer 5'UTR, a wide range of 5' and 3' UTR length as compared to Arabidopsis and rice (Figure 1h). They should mention median lengths in each organism in the paper. Is the observed difference statistically significant? (not shown). Also, it is not clear the source of these 5' and 3'UTR lengths of rice and Arabidopsis as there are not any systematic studies on UTRs. They should provide details on the source of these data.
EMSA assay – No details are provided as to what region of the promoter (and its length) was used in this assay.
Nitrate uptake studies with HEK 293 cells - Is the uptake shown with L and S form is after subtracting the nitrate uptake in control cells (with empty vector)? It is not clear from the methods and legends. The figure 2h should show nitrate uptake with empty vector.
Generally, global RNA-seq studies are done in triplicates. All RNA-seq studies reported here are done duplicates.
Suppl Fig .2 b-f: show the examples shown for splice junctions. None of them correspond to canonical junctions. Is every one of them has non-canonical splice junctions? Explain
Suppl Fig 1e bottom panel – I am assuming this is read depth? What do those numbers in the bottom represent? Do they represent reads that span splice junctions?
None of the supplementary tables have titles. It would be helpful for the reader to include titles.

Reviewer #3 (Remarks to the Author):

Key Results: Continuing their previous efforts in sequencing the cotton genome, in this work the authors presented a large study of the cotton transcriptome for genome annotation. By using four sequencing experiments to analyze transcription initiation, termination and splicing from multiple tissues, combined with a careful informatics pipeline, the authors predicted a large portion of the cotton transcriptome and QA/QC a subset with independent experiments. The resulting transcriptome was then cataloged for various features including tissue-specific transcription, alternative transcription initiation and termination, regulated alternative splicing (micro-exons, read-throughs), etc.

Validity: The work described in the manuscript is overall sound, with major conclusions supported by the data.

Originality and significance: The work appears to be original, and it provides an important resource for the plant genomics research community.

Data & methodology: The raw data and software pipelines have been made clearly documented. I was not able to locate the raw data from the NCBI SRA, I assume it is under embargo? The annotations (isoforms, TSSs, TESs, etc) need to be publicly available, ideally in some forms of databases and genome browsers (such as cottongen), so that the plant research community would benefit from this work.

Suggested improvements:
1. It is not clear to me from reading the main text or the supplemental materials (no supplemental text, just tables and figures), how the RNA-Seq libraries were constructed. My impression was size-selected polyA RNA was used, please confirm. If this is the case, it should help to clarify that many noncoding RNAs, small RNAs or circular RNAs were not analyzed in this study. This is not a weakness, just need some clarification.
2. The sequencing depth of PacBio seems to be critical for predicting isoforms based on how the informatics pipleline works. On L99 the authors stated "close to saturated depth", but given on L114 a total of "36,826 genes" were predicted, on supplemental Figure 1b it appears less than 30,000 genes were detected by the PacBio reads. It seems to me that >20% of the genes were not hit by any PacBio reads, let alone their isoforms, is that right? Please clarify.
3. L75: "transcript isoforms are full of errors[22]", consider revising to be precise.
4. L106-108, I was under the impression that the IGIA core isoforms are solely from PacBio reads in regions with PacBio coverage (not from TACO). If this is the case, for regions that PacBio sequencing does not have sufficient coverage (see comment #2), we would miss rare isoforms.

References: The author should cite ToFU (Gordon et al., 2015). Also, cite this for the discovery of polycistronic/read-through RNA.

Clarity and context: I could follow the majority of the manuscript, but there are numerous grammar errors. I suggest the manuscript to be language edited to be more concise and clearer.

Reviewer #4 (Remarks to the Author):

Cotton is the leading crop for renewable fibers, and the fiber has been long focused by the community of agronomist and plant cell and molecular biologist. As the extent diploid progenitor of modern tetraploid cultivated cottons, Gossypium arboreum, bearing spinnable fibers, is an ideal model for fiber development. The MS described the global identification and validation of variations in G. arboreum transcriptome in various tissues. The abundant data were reliable, providing a good platform for further researches on fiber biology. To increase the importance of the data in fiber biology, several points need to be improved in the revision.
Fiber is unique single cell for its length and chemical composition. In this manuscript, the developing seeds and fibers are sampled in mixture to obtain the transcriptomic data. To construct the fundamental data platform for fiber biology, the authors are encouraged to sample the developing fibers and ovules separately. Technically, the isolation of RNA from the early stage fibers from their matrix (i.e., ovule) is no longer an obstacle for this work. For example, EW Taliercio and D Boykin developed a method to isolate RNA from fiber initials (BMC Plant Biology, 2007, 7:22 doi:10.1186/1471-2229-7-22). Using mixture RNAs, the dynamic and diverse transcriptional phenomena (such as alternative usage of transcription start sites, and alternative polyadenylation and polycistrons) described in the manuscript contain the information from both fiber and seed, and some important information unique in the fiber may be eclipsed. Thus, using fiber RNA to construct transcriptomic data base, I believe, is reasonable, which can provide valuable information for searching of bona fide genes involved in fiber development, or associated with fiber yield/quality.
Only RT-PCR was used to confirm the role of candidate genes. It’s better to add some transgenic,

biochemical or physiological evidences.
In addition, as a fundamental data or tool for further researches, the results should be easily accessible, like in JBrowse or in JGI (https://phytozome.jgi.doe.gov). Furthermore, I suggest authors to compare their results with the related data of G.raimondii and G. hirsutum in JGI.

Point-by-point responses:

Reviewer #1 (Remarks to the Author):

In this manuscript, the authors have essentially sought to integrate a variety of sequencing data modalities including Iso-seq, ssRNA-seq, CAGE-seq and PolyA-seq to extensively characterize the transcriptome landscape of Gossypium arboreum, a diploid cotton genome. In addition to tissue-specific gene expression, they investigated features such as transcription start sites, alternative polyadenylation, alternative splicing events and SNPs including those outside coding regions. They have devised an approach IGIA (integrative gene isoform assembler) that allows a better integration of PacBio Iso-seq and NGS based ssRNA-seq, CAGE-seq and polyA-seq than any other existing tools. More specifically, they have demonstrated how the integrative/hybrid approach performs better than ToFU for long reads or TACO, StringTie, Cufflinks etc for short reads. This work improves and extends the existing resources such as Cottongen and CGP in the context of G. arboreum and is certainly of value to the cotton community.

We appreciate the reviewer for the positive comments and agreeing that the results are of value to the cotton community.

1. The manuscript mentions a number of bioinformatics tools that authors have used for a variety of analyses: RAMPAGE, CPAT, proovread, KAAS, QGRS Mapper, rMATS, ANCHOR, GoSemSim, iupred, VSL2, anchor, signalP, TMHMM and many others. It will be helpful to the readers to have a listing of all the referred tools, along with a brief description of their main functionality, related publication and download site.

Response: Thank you for the suggestion. We have compiled a list with the information and inserted it in **Supplementary Table 20**. We have also added a description in "Code availability" in the Methods section (Line 739) as follows: "All the tools used in this study are listed in **Supplementary Table 20**".

2. The authors have used sound statistical analyses throughout the paper. However, the authors use various values of cutoff for FPKM, TPM etc without explaining the underlying rationale for the choice of cutoff. For example, FPKM >5 when comparing the different approaches for predictions of splicing junction sites; FPKM >0.5 and 0.2 for single and multi-exon fragments respectively to filter out low expressed fragments; average TPM >0.5 to identify TSS and TES selection.

Response: We thank the reviewer for this suggestion. Per your comment, a detailed description explaining the underlying rationale for the choice of cutoff values has now been added to the Results and Methods section.

(1) Our initial idea was to validate the JS of genes with FPKM values >5 for higher transcript levels and then compare the accuracy of JS annotations for CGP and IGIA as

well as Cottongen and IGIA. However, after careful consideration, we thought that validation with only the higher expression genes (FPKM >5) may affect the objectivity of the evaluation results due to the differences in sensitivity of various annotations from IGIA, CGP, and Cottongen. Therefore, in the revised manuscript, we further expanded the scope of the verification, providing more gene examples with FPKM <5, and finally allowing our validation to cover a wider range of gene expression. Genes were divided into four quantiles based on sorted FPKM values: 0–25%, 25–50%, 50–75%, and 75–100%. These validation examples were added in **Supplementary Table 5**, and the corresponding description in Results (Line 134) and **Supplementary Figure 2** was revised.

(2) To complement the IGIA core isoforms and actively annotate transcriptional regions as much as possible, TACO was used to identify the missed transcripts. Because the TACO pipeline has very high sensitivity, a considerable number of noise signals in cotton genome would be annotated as transcripts. Based on our expression analysis (using FPKM values) of single-exon and multi-exon fragments identified in TACO (**Supplementary Figure 12c,d**), FPKM = 0.5 was chosen as the cutoff value to filter approximately 50% of lowly expressed single-exon fragments (**Supplementary Figure 12c**). Moreover, since multi-exon fragments have longer length and higher average expression, FPKM = 0.2 was chosen as the cutoff value to filter approximately 5% of multi-exon fragments (**Supplementary Figure 12d**).

The goal of the above treatment was to retain reliable signals as much as possible and obtain more comprehensive annotations. We have added the explanation for the choice of cutoff values in the subtitle “TACO pipeline” in the Methods section (Line 584).

(3) For the cutoff value (TPM = 0.5) used in TSS and TES selection, we cited the protocol paper and added description in the part of “Alternative TSS and TES function analysis” in the Methods section (Line 628).

3. Lines 45-46: Thus, the cotton is one of the most economically important crop plants world-wide and has long been one of major focuses of plant research [1, 2].
>> Cotton is unarguably a very important crop on a global scale but it does not belong to the group of most valuable crops economically or say in production metric, with rice, wheat, soybean, tomatoes, sugarcane, maize, potatoes and grapes well ahead of it in financial global value. For more specific global statistics, authors are encouraged to visit website of the Food and Agriculture Organization of the United Nations. Cotton is, however, one of the most economically important fiber crop plants world-wide.

Response: Thank you for your comment. We have now changed the sentence in the revised manuscript as follows: “Thus, cotton is one of the most important fiber crop plants worldwide and has long been a major focus of plant research”.

4. Lines 47-50: In the past few years, four different cotton genomes have been successfully sequenced and assembled, including two allotetraploid (AADD) Gossypium hirsutum[3, 4, 5] and Gossypium barbadense[5] genomes, and two ancestor

diploid Gossypium raimondii (DD)[1, 6] and Gossypium arboreum (AA)[7, 8].
>> Levant cotton (Gossypium herbaceum) is another genome that has been undergone transcriptomic profiling in recent years and is in fact a close relative of Gossypium arboreum.

Response: Thanks. We have added the description about transcriptome profiling in Levant cotton and corresponding reference[10] to the Background section.

5. Lines 79-81: A computational method named GRIT reconstructed gene models from RNA-seq short reads data by integrating CAGE-seq and PolyA-seq data for Drosophila genome, and has obtained better results than Cufflinks based only on RNA-seq data[26].
>> The authors should discuss GRIT in further details, since one of the main product of this manuscript, IGIA, is being advocated as superior to GRIT. The authors should also discuss how IGIA compares with GRIT, both in conceptual design as well as accuracy/performance metric.

Response: Thanks for the suggestion. The GRIT algorithm is suitable for integrating NGS-based ssRNA-seq, CAGE-seq, and PolyA-seq data, which cannot support long reads data from Pacbio-seq. Therefore, its accuracy/performance metric with our data was not evaluated. We have added the discussion regarding the similarities and differences in conceptual design between GRIT and IGIA in the second paragraph of the Discussion section (Line 430).

6. Lines 112-113: Integrating the IGIA core isoforms (IsoF and IsoR) and IsoC, and those isoforms from our TACO pipeline
>> Drop ‘our’ from ‘our TACO pipeline’, unless one or more of the authors have contributed to the development of TACO pipeline.

Response: We have corrected the sentence. We used TACO in our customized pipeline, thus we have dropped “our” and changed to “adjusted TACO pipeline”.

7. Line 122: The cumulative fraction curves show that the transcripts from ToFU annotations with Pacbio long reads

Response: We apologize for missing the reference. It has been added in the revised manuscript.

8. Lines 223-224: Next we analyzed the consequences on protein for a set of 2,800 proteins with alternative TSSs in coding regions.
>> It is unclear what the authors mean by “consequences on protein“.

Response: We modified the text to read “Next, whether alternative TSSs in 2,800 genes would change their protein sequences was investigated”.

Here are a few minor edits:

9. Lines 53-55: Accumulating evidence supports that the regulation of alternative transcript isoforms plays pivotal functions on eukaryote development ...
>> … regulation of alternative transcript isoforms plays pivotal role in eukaryote development ...

Response: Corrected.

10. Lines 61-62: The regulation of RNA transcription and processing significantly thus affects multiple aspects …
>> The regulation of RNA transcription and processing thus significantly affects multiple aspects …

Response: Corrected.

11. Lines 361-362: These bicistronic mRNA is produced by transcriptional read-through for the two adjacent genes.
>> This bicistronic mRNA is ...

Response: Corrected.

12. Lines 450-451: However, the biogenesis and functions of AS hotspot in plants, and same questions to polycistrons, are both intriguing issues to be investigated in future studies.
>> It is unclear what authors mean by 'and same questions to polycistrons'

Response: We meant the questions about biogenesis and functions of polycistrons. We have revised the sentence to make it clear.

13. Lines 492-493: The 5' random barcode in Read1 and Read2, polyA stretch in Read1 and polyT stretch in Read2 were trimed.
> 'trimed' is misspelled.

Response: Corrected.

14. Lines 523-525: For reads that can not perfect with all of the above methods, we will try to improve it by enumerate its exon path like other NGS-based methods, and the enumerated isoform is called Partial information isoform (isoP).
>> The intent of this sentence is not clear. Consider rephrasing it for clarity.

Response: We have modified the text as the following: For a long read with unsupported junctions not rescued by the above methods, we removed those wrong junctions and filled the gaps by enumerating exons at the loci identified from NGS

RNA-seq; the resulted isoform was termed as partial isoform (isoP).

15. Lines 577-579: A gene which has at least two dominated TSS site and the change of which usage rate greater than 30% of these two site in two tissues is called dynamic TSS swtich gene.
>> Once again, the intent of this sentence is not clear. Consider rephrasing it for clarity. Also, 'TSS swtich gene' is misspelled.

Response: Thanks for the suggestion. We have modified the text to make it clear: For a gene with multiple TSSs, the dominated TSS in one tissue was defined as the TSS having more than 50% CAGE-seq signal across the gene. In analyzing differential TSS usage across multiple tissues, a dynamic TSS switch gene was identified by the two criteria: it has two or more dominant TSSs in any two tissues and the TSS switch score is >0.3. The scoring formula was added in the Method section (Line 633).

Reviewer #2 (Remarks to the Author):

The authors of this manuscript have used four different sequencing methods (PacBio iso-seq, RNA-seq, CAGE-SEQ and polyA-seq) to analyze the landscape of a diploid cotton G. arboretum transcriptome using RNA from sixteen different tissues. <u>The main contribution of this paper is the annotation of transcripts in this species including 5'UTRs, 3'UTRs with alternative TSS/TES and alternative splicing. The major conclusions of this paper are convincing, but several of their conclusions are confirmatory</u>. For example, a recent global analysis of alternative splicing, poly(A) and fusion transcript analysis using PacBio Iso-seq in another cotton species (Wang et .al., New Phytol. 2018) and other global alternative splicing papers have reported similar conclusions. Alternative splicing in other diploid and tetraploid species has also been reported (Li et al., Mol. Plant 2014; Zhu et al., BMC Genomics 2018). <u>However, the transcription sites (TSSs) in cotton transcripts have not been reported and this paper adds new information in this area. The information presented in this paper, especially the updated gene/transcript annotation would be useful to the researchers in the cotton community particularly those that are working with the diploid species G. arboretum.</u>

We thank the reviewer for the positive comments to our manuscript.

1. I think an interesting topic that could be addressed with the data that the authors have is the relationship between alternative transcription start sites and alternative splicing as well as alternative splicing and transcription end sites. Analysis of their data to address if there is any coupling between these processes would add novelty to this paper and appeal to the broader research community. Also, some comparative analysis of finding on AS and poly(A) results in this work with recently reported results in another cotton paper (Wang et al., New Phytology 2018) should be presented in the discussion.

Response: Thank you for your suggestions. We have now added the analysis of the

relationship between alternative transcription start sites (TSSs) and alternative splicing as well as alternative splicing and transcription end sites (TESs). We found many coupling events between those processes. The corresponding analysis results have been added in **Supplementary Figure 10a-d**, and described in Results section (Line 345). Additionally, we have added discussion to compare the findings on AS and Poly(A) with recently reported results in an allotetraploid cotton (Wang et al., New Phytology 2018) (Line 486).

2. It was stated that if the TSS clusters are closer to each other (with in 400bp), only the TSS position with the largest signal was chosen as TSS candidates for integration. They applied the same criteria for TES clusters also. What is the basis for this cutoff? Does this underestimate alternate TSSs and TESs?

Response: We have now explained the basis for this cutoff (400 bp) in "IGIA pipeline" subsection of Methods (Line 552), which was based on a statistical analysis. In fact, we only used this cutoff in gene annotation to simplify the gene models. For the analysis of alternative TSS and TES events, the cutoff 50 bp was used (see "Alternative TSS and TES function analysis" in the Methods section, Line 631). Thus, alternative TSSs and TESs in our analysis were not underestimated.

3. The authors conclude that G. arboretum genome has unique features including longer 5'UTR, a wide range of 5' and 3' UTR length as compared to Arabidopsis and rice (Figure 1h). They should mention median lengths in each organism in the paper. Is the observed difference statistically significant? (not shown). Also, it is not clear the source of these 5' and 3'UTR lengths of rice and Arabidopsis as there are not any systematic studies on UTRs. They should provide details on the source of these data.

Response: As requested, we have now mentioned the median lengths and statistical significances in **Figure 1h**. We added the source of these data in **Supplementary Table 1**. All the information of the gene structure was from the updated genome annotation data (e.g., rice from MSU 7.0 and *Arabidopsis* from Araport 11). When we performed the analysis for the genes with 5' UTRs and 3' UTRs, the genes without UTRs were excluded.

4. EMSA assay – No details are provided as to what region of the promoter (and its length) was used in this assay.

Response: We appreciate your comment. We have now provided the sequences and length of DNA probes in the **Supplementary Table 5** of the revised manuscript. We have also added the description of EMSA assay in the Methods section (Line 701).

5. Nitrate uptake studies with HEK 293 cells - Is the uptake shown with L and S form is after subtracting the nitrate uptake in control cells (with empty vector)? It is not clear from the methods and legends. The figure 2h should show nitrate uptake with empty

vector.

Response: Yes, the uptake data in **Figure 2h** were the difference of measured values of NRT-L and NRT-S minus that of control (with empty vector), respectively. As suggested, we have modified the histogram in **Figure 2h** by adding nitrogen uptake data from the empty vector. The **Figure 2h** legend was also modified accordingly.

6. Generally, global RNA-seq studies are done in triplicates. All RNA-seq studies reported here are done duplicates.

Response: We agree with the Reviewer that triplicates would be more helpful for evaluating data reproducibility. We also would like to point out that, based on ENCODE guidelines (www.encodeproject.org), researchers generally perform duplicates for RNA-seq studies, as in recent studies on maize (*Plant Cell* 2019, 31:974) and wheat (*Science* 2018, 361:662). In our case, our two ssRNA-seq libraries from two independent experiments have shown a high reproducibility in all samples, including the newly obtained ones during revision (**Supplementary Figure 4**). Moreover, the data among 16 tissues are cross-validated with each other, as the obtained sensible relationships shown in **Supplementary Figure 5a** and **6b**. The specialized CAGE-seq and PolyA-seq data from 16 tissues are also cross-validated with corresponding ssRNA-seq data, as that shown in **Figures 2e** and **3i**, and **Supplementary Figure 10c and 10d**. Thus, we believe that our current RNA-seq settings are sufficient to support our conclusions.

7. Suppl Fig .2 b-f: show the examples shown for splice junctions. None of them correspond to canonical junctions. Is every one of them has non-canonical splice junctions? Explain

Response: Thanks for the question. Not all of them were non-canonical splice junctions. For CGP and Cottongen annotations, approximately 54% (46/85) and 66% (59 /89) of error annotations occurred in canonical junctions. We have added the statistics of experimental validations in **Supplementary Table 5**.

8. Suppl Fig 1e bottom panel – I am assuming this is read depth? What do those numbers in the bottom represent? Do they represent reads that span splice junctions?

Response: The numbers represent the reads that span splice junctions. We have now added the details in the Figure legend.

9. None of the supplementary tables have titles. It would be helpful for the reader to include titles.

Response: Thanks for the suggestion. We have added titles for all the supplementary tables.

Reviewer #3 (Remarks to the Author):

Key Results: Continuing their previous efforts in sequencing the cotton genome, in this work the authors presented a large study of the cotton transcriptome for genome annotation. By using four sequencing experiments to analyze transcription initiation, termination and splicing from multiple tissues, combined with a careful informatics pipeline, the authors predicted a large portion of the cotton transcriptome and QA/QC a subset with independent experiments. The resulting transcriptome was then cataloged for various features including tissue-specific transcription, alternative transcription initiation and termination, regulated alternative splicing (micro-exons, read-throughs), etc.

Validity: The work described in the manuscript is overall sound, with major conclusions supported by the data.

Originality and significance: The work appears to be original, and it provides an important resource for the plant genomics research community.

We thank the reviewer for the positive comments on the validity, originality, and significance of our work.

Data & methodology: The raw data and software pipelines have been made clearly documented. I was not able to locate the raw data from the NCBI SRA, I assume it is under embargo? The annotations (isoforms, TSSs, TESs, etc) need to be publicly available, ideally in some forms of databases and genome browsers (such as cottongen), so that the plant research community would benefit from this work.

Response: Thanks for the positive comment. Yes, the raw data deposited in SRA are under embargo. We have included the link to view the raw data (https://dataview.ncbi.nlm.nih.gov/object/PRJNA507565?reviewer=lcb97hmjcukj5pucaj13h5mml1). We have developed a genome browser with our annotations and made the data publicly available (http://cotton.whu.edu.cn/igia). Thanks for the suggestion.

Suggested improvements:
1. It is not clear to me from reading the main text or the supplemental materials (no supplemental text, just tables and figures), how the RNA-Seq libraries were constructed. My impression was size-selected polyA RNA was used, please confirm. If this is the case, it should help to clarify that many noncoding RNAs, small RNAs or circular RNAs were not analyzed in this study. This is not a weakness, just need some clarification.

Response: We apologize for having left out the information about RNA-seq library construction. We used "Ribo-Zero rRNA Removal Kit" to deplete ribosome RNAs, and

then constructed the ssRNA-seq libraries according to referenced protocol (Parkhom *et al.*, 2009) with size-selection of inserts for sequencing. The insert lengths were around 200 bp (sd = 40 bp), which explained no small RNAs were detected. In fact, our IGIA gene set contained many non-coding genes. However, we only focused on analyzing the coding genes in this study. As suggested, we have now clarified it in the revised manuscript. We have also included the description of RNA-seq library construction and corresponding reference in the Methods section (Line 515).

2. The sequencing depth of PacBio seems to be critical for predicting isoforms based on how the informatics pipeline works. On L99 the authors stated "close to saturated depth", but given on L114 a total of "36,826 genes" were predicted, on supplemental Figure 1b it appears less than 30,000 genes were detected by the PacBio reads. It seems to me that >20% of the genes were not hit by any PacBio reads, let alone their isoforms, is that right? Please clarify.

Response: We appreciate your comment and have described this more accurately in the revised manuscript. In this project, the sizes of Pacbio libraries were <1 kb, 1–2 kb, 2–3 kb, and >3 kb, respectively. The observed median lengths of reads of insert (ROI) for the four groups were 1,048, 1,854, 2,847, and 4,174 nt, respectively (**Supplementary Table 1**), indicating that the shorter transcripts were not well captured in Pacbio libraries. We counted the number of genes with or without Pacbio read supports based on library size. As expected, nearly 50% of the genes <1 kb were not covered by Pacbio reads, which accounted for 58% of the genes not hit by Pacbio. In addition, for genes 1–3 kb and >3 kb in length, Pacbio reads covered 85% and 95% of genes, respectively. In summary, for long genes, current Pacbio sequencing depth is close to saturation. For short genes, the splicing modes were expected to be simple, thus, would not heavily depend on Pacbio long reads. We have now clarified these in the subsection “IGIA and TACO integration” of Method section (Line 597).

3. L75: "transcript isoforms are full of errors [22]", consider revising to be precise.

Response: Thanks. We have revised the text to be more precise.

4. L106-108, I was under the impression that the IGIA core isoforms are solely from PacBio reads in regions with PacBio coverage (not from TACO). If this is the case, for regions that PacBio sequencing does not have sufficient coverage (see comment #2), we would miss rare isoforms.

Response: Yes, you are correct. The core IGIA annotation might miss rare isoforms, which were recovered in our complete IGIA annotation set. The regions without Pacbio long read coverage are generally due to low gene expression and short gene length (**Figure 1e**). In this study, we aimed to make a gene set with stronger evidences. The IGIA core annotation set is more reliable, and the complete annotation set is more comprehensive. We have added this point to the Discussion section (Line 437). Thanks.

5. References: The author should cite ToFU (Gordon et al., 2015). Also, cite this for the discovery of polycistronic/read-through RNA.

Response: Thanks for the suggestion. We have added the ToFU citation [30] in the Methods section and in the result section of "Discovery of polycistrons and their genomic features".

6. Clarity and context: I could follow the majority of the manuscript, but there are numerous grammar errors. I suggest the manuscript to be language edited to be more concise and clearer.

Response: Thanks for the suggestion. The revised manuscript has been edited by a native English speaker.

Reviewer #4 (Remarks to the Author):

Cotton is the leading crop for renewable fibers, and the fiber has been long focused by the community of agronomist and plant cell and molecular biologist. As the extent diploid progenitor of modern tetraploid cultivated cottons, Gossypium arboreum, bearing spinnable fibers, is an ideal model for fiber development. The MS described the global identification and validation of variations in G. arboreum transcriptome in various tissues. The abundant data were reliable, providing a good platform for further researches on fiber biology. To increase the importance of the data in fiber biology, several points need to be improved in the revision.

We thank the reviewer for the positive comments to the methods and the data generated in our study.

1. Fiber is unique single cell for its length and chemical composition. In this manuscript, the developing seeds and fibers are sampled in mixture to obtain the transcriptomic data. To construct the fundamental data platform for fiber biology, the authors are encouraged to sample the developing fibers and ovules separately. Technically, the isolation of RNA from the early stage fibers from their matrix (i.e., ovule) is no longer an obstacle for this work. For example, EW Taliercio and D Boykin developed a method to isolate RNA from fiber initials (BMC Plant Biology, 2007, 7:22 doi:10.1186/1471-2229-7-22). Using mixture RNAs, the dynamic and diverse transcriptional phenomena (such as alternative usage of transcription start sites, and alternative polyadenylation and polycistrons) described in the manuscript contain the information from both fiber and seed, and some important information unique in the fiber may be eclipsed. Thus, using fiber RNA to construct transcriptomic data base, I believe, is reasonable, which can provide valuable information for searching of bona fide genes involved in fiber development, or associated with fiber yield/quality.

Response: We appreciate your comment. In the revised manuscript, we have applied our method (Shi, et al., Plant Cell, 2006, 18:651) to strip the epidermal fibers from ovules at 5, 10, and 20 DPA, and then performed ssRNA-seq with two biological replicates of fibers and ovules (without fibers), separately. We have performed thorough analysis and constructed the transcriptome map of developing fibers. We added **Supplementary Figure 6** and description to present the corresponding results in the Results section (Line 194). We also included the fiber-specific genes in **Supplementary Table 8.** Those new results provide valuable information for researchers in fiber biology.

2. Only RT-PCR was used to confirm the role of candidate genes. It's better to add some transgenic, biochemical or physiological evidences.

Response: Thanks for the suggestion. In the revised manuscript, we have validated fiber-specific genes with qPCR and *in situ* hybridization assay (**Supplementary Figure 6**). For other candidate genes, we performed EMSA assay to confirm binding affinity change of AP2 caused by regulated microexon splicing, and also performed nitrogen transport assay to verify the activity change of NRT1.2 due to TSS switch. We agree with the suggestion about the transgenic study, and this belongs to a follow-up study of our current system-wide transcriptomic analysis.

3. In addition, as a fundamental data or tool for further researches, the results should be easily accessible, like in JBrowse or in JGI (https://phytozome.jgi.doe.gov). Furthermore, I suggest authors to compare their results with the related data of G.raimondii and G. hirsutum in JGI.

Response: Thanks for the suggestions. We have developed a genome browser (http://cotton.whu.edu.cn/igia) with our results including IGIA gene annotation and all RNA signal tracks of Pacbio-seq, ssRNA-seq, CAGE-seq, and PolyA-seq data. This web server is freely accessible to the world-wide researchers. The link has been added to the "Data Availability" section in the revised manuscript (Line 743). Furthermore, we have compared our gene annotations with *G. raimondii* and *G. hirsutum* in JGI (please see revised **Figure 1h**).

REVIEWERS' COMMENTS:

Reviewer #1 (Remarks to the Author):

My own prior review as reviewer #1: I would like to commend the authors for having sufficiently and satisfactorily addressed the clarifications, corrections and revisions including a new supplementary table that I requested in the original review. I believe the end product is a manuscript that is robustly supported by data and more consistent and comprehensive.

Additional comments regarding the value of this work are available in my original review.

(NEW) Review for comments from reviewer #2: I believe the authors have sufficiently addressed all comments from this reviewer. The authors have done sufficient due diligence with updates in tables, figures, legends and revised text to address the missing links in explanations.

One of the weaknesses of this study is using duplicates instead of triplicates (as this risks lower statistical power and reproducibility). However, with the data in hand, the authors have tried to satisfy this concern by providing cross-validations as needed.

Reviewer #2 unavailable.

Reviewer #3 (Remarks to the Author):

All my previous concerns are adequately addressed. I have no further comments.

Reviewer #4 (Remarks to the Author):

The questions in the previous submission has been well addressed.
I think that the present MS is acceptable.
